# Navigating the medical journey: Insights into medical students’ psychological wellbeing, coping, and personality

**DOI:** 10.1371/journal.pone.0318399

**Published:** 2025-02-06

**Authors:** Aisha Ali Hawsawi, Neil Nixon, Elena Nixon

**Affiliations:** 1 Institute of Mental Health, Mental Health and Clinical Neurosciences, School of Medicine, University of Nottingham, Nottingham, United Kingdom; 2 School of Education, Health and Behavioral Sciences, Dar Al-Hekma University, Jeddah, Saudi Arabia; 3 Nottinghamshire Healthcare NHS Foundation Trust, Nottingham, United Kingdom; University of Macerata: Universita degli Studi di Macerata, ITALY

## Abstract

**Background and aims:**

In recent years, increased awareness of the psychological wellbeing of healthcare professionals and students has become a pressing public health issue affecting care delivery. Medical students undergo rigorous training programs that can affect their psychological wellbeing. Despite increased awareness of mental health issues among medical students, research often focuses on negative aspects, overlooking potential positive contributors to wellbeing. This study aims to explore both negative and positive factors influencing medical students’ psychological wellbeing, considering coping strategies and personality traits to inform targeted support measures for diverse student needs.

**Methods:**

A mixed-methods approach was employed to investigate medical students’ psychological wellbeing, coping strategies, and personality traits. Quantitative data was gathered via self-report questionnaires and analysed using regression models. Additionally, qualitative insights were obtained from semi-structured interviews and analysed thematically to capture students’ perceptions and experiences.

**Results:**

The analysis revealed moderate to high levels of stress, anxiety, and depression among medical students, along with decreased life satisfaction. Regression analysis showed that problem-focused coping positively impacted medical students’ psychological wellbeing, whereas emotion-focused and avoidance coping showed less favourable effects. Notably, problem-focused coping partially mediated the relationship between stress and depression. Furthermore, personality traits, particularly agreeableness and conscientiousness, played a pivotal role in shaping medical students’ coping strategies and mental health outcomes. Based on thematic analysis, codes gave rise to three overarching themes and corresponding subthemes.

**Conclusions:**

The study underscores the significance of addressing both positive and negative factors impacting medical students’ wellbeing and highlights the need for tailored support considering individual personality traits that influence coping strategies and mental health. It also identifies challenges within medical education, emphasising the necessity for stress management programs, mental health support, and curricula promoting problem-solving skills. Prioritising medical students’ wellbeing may not only foster good mental health among future professionals but may also enhance future healthcare quality.

## Introduction

The global acknowledgement of challenges to the mental health and psychological wellbeing of medical students has been intensified within clinical, educational, and research contexts [1,2]. Promoting students’ wellbeing has become an essential institutional objective for cultivating competent physicians and optimising patient care [3,4]. However, navigating medical education presents numerous hurdles to medical students’ psychological wellbeing, as evidenced by previous research [5,6]. In a study by Yusoff et al. [5] looking at stressors experienced by medical students, academic, psychosocial, environmental, and financial stressors were identified; these stressors were rated as causing moderate to high stress levels, with academic stressors having been significantly associated with distress. Additionally, Hill et al. [6] corroborated these findings, emphasising the impact of workload, study-life balance, and pre-existing mental health disorders on medical students; students also expressed concerns about academic demands, long work hours during placements, performance pressure, external stressors -like board exams- and competing responsibilities. Conversely, the social support networks essential for the wellbeing of medical students are often compromised, leaving students with limited opportunities for recuperation [3,6]. Moreover, the COVID-19 pandemic has exacerbated the situation, disrupting medical education through remote learning, cancelling clinical rotations, and heightening uncertainty [7].

The aforementioned stressors, along with the pandemic, have contributed to a pronounced decline in the mental wellbeing of medical students, marked by manifestations of high stress levels, depression, anxiety, burnout, suicidal ideation, and increased substance use [8,9]. A recent systematic review and meta-analysis has shed light on the alarming prevalence of depression among medical students, estimating rates to be three times higher than in age-matched groups, with an unexpected one in three students reporting symptoms of anxiety [10]. Multiple studies have highlighted a worrying trend: medical students start their education with psychological wellbeing comparable to the general population but experience significant increases in depression and anxiety during their studies [11,12], resulting in decreased life satisfaction too [13,14]. This evidence highlights a noteworthy shift in mental health dynamics, emphasising the pivotal role of medical education in influencing the psychological wellbeing of its students.

The significance of medical students’ mental health extends beyond its prevalence, as it has the potential to impede the acquisition of competencies, impair decision-making, compromise patient interactions, and adversely affect overall performance [8,15]. Additionally, untreated mental health issues in medical students may serve as a foundational factor contributing to the poor mental health observed among physicians [16]. Therefore, addressing the mental health of current medical students is imperative to ensure the wellbeing of future healthcare professionals.

In confronting the challenges presented by stressors and their impact on psychological wellbeing, recent research has underlined the necessity to advocate for the adoption of effective coping strategies among medical students. This emphasis arises from the recognition that coping strategies act as calming and alleviating elements, playing a crucial role in empowering individuals to maintain a mental balance amidst stressful circumstances [17,18]. Consequently, the adapted use of coping strategies not only helps alleviate stressors but also nurtures sustained psychological resilience and wellbeing in the long term for medical students [19,20]. These coping approaches, which include problem-focused, emotion-focused, and avoidant coping strategies, are critical considerations for medical students. Their outcomes may involve dysfunctionality or functionality, respectively elevating or diminishing the likelihood of burnout [21,22]. Steiner-Hofbauer and Holzinger [23] supported this evidence, suggesting that active coping, including planning and positive thinking, is linked to lower stress levels and acts as a protective factor against depressive symptoms. Additionally, Park and Adler [24] found that medical students who utilised problem-focused coping strategies experienced less deterioration in both physical health and overall wellbeing. Building upon the discourse on coping strategies, research findings have illuminated the intricate interplay between individual coping styles and personality traits, such as the “Big Five” personality traits—neuroticism, extraversion, openness to experience, conscientiousness, and agreeableness [17,25]. Such findings have shed light on how individual coping styles are influenced by these traits, providing valuable insights into the intricate dynamics shaping psychological wellbeing.

Afshar et al. [25] and Gramstad et al. [26] showed that individuals high on neuroticism often resort to emotion-oriented coping or disengagement strategies to alleviate the intensity of emotional reactions to stress. Conversely, extroverted individuals, as outlined by Connor-Smith and Flachsbart [17], lean towards adopting problem-solving strategies and seeking instrumental support as coping strategies.

Furthermore, conscientious individuals, tend to demonstrate a focus on organising plans to mitigate the impact of stress [27]. Agreeable individuals, as highlighted by Chen [28], are inclined to seek social support and engage in cognitive restructuring as coping strategies. Additionally, openness to experience has been associated with positive reappraisal, seeking information, and problem-solving, and has been found to have a negative correlation with avoidance coping [17].

While the literature has increasingly addressed the psychological wellbeing of medical students [1,9,16,29], it has primarily focused on negative factors [30–34], neglecting positive aspects such as social support, quality of life, and resilience [35–38]. Identifying these factors early in medical training is crucial for promoting current and future wellbeing, as well as informing tailored support interventions.

This study aims to explore potential positive contributors to medical students’ psychological wellbeing during their medical training. The research adopts the theoretical framework of the Dual Continuum Model of mental health and illness (Westerhof & Keyes, 2010) [85], which emphasizes the importance of considering both positive mental health and the absence of mental illness. Aligned with the principles of positive psychology, the study recognises that individual strengths, positive experiences, and other favourable attributes play a significant role in promoting optimal functioning and wellbeing. Therefore, alongside addressing negative aspects of wellbeing, it aims to identify and enhance factors that contribute positively to the lives of medical students, fostering resilience and facilitating personal and professional growth. This study hence adopts a mixed-methods approach to gain a comprehensive understanding of factors affecting medical students’ psychological wellbeing, aiming to capture both measurable and contextual dimensions of mental health within this population. The quantitative analysis focuses on examining levels of stress, depression, anxiety, and life satisfaction in relation to specific coping strategies and personality traits, investigating how these factors interact in ways that influence wellbeing. The qualitative component complements this investigation by exploring students’ personal experiences and perceptions, particularly how they navigate stressors in both controllable and uncontrollable situations, such as the academic pressures and the impact of the COVID-19 pandemic. In contrast to previous studies that have examined these aspects separately [1,9,16,29], this paper combines quantitative and qualitative insights. This combination offers a fresh perspective on how personality traits may influence the effectiveness of coping strategies and, in turn, may affect mental health outcomes.

## Materials and methods

### Study design

Medical students from Nottingham University were recruited for the study, which employed a mixed methods design; quantitative data was collected through an online survey, utilising the JISC Online Survey platform. The survey encompassed a demographic form and five questionnaires assessing stress, anxiety, depression, life satisfaction, coping strategies and personality traits (see details in *Measures*). To adhere to COVID-19 ethics regulations, remote interviews using Microsoft Teams were conducted. Subsequent to completing the questionnaires, participants expressing interest were invited to participate in the remote semi-structured interviews (see details below in Measures).

### Participants

This study largely recruited participants during the COVID-19 pandemic. G power was employed as the calculation method to establish the sample size, with a 5% margin of error and a 95% confidence level. The estimated sample size for completing the questionnaires was n = 250 medical students. For the qualitative component involving semi-structured interviews, approximately 25 medical students were sought. This number was determined based on established protocols and methodologies, particularly those employing thematic analysis [39]. Medical students from undergraduate and graduate programs were invited to participate to ensure diverse perspectives and experiences.

*Inclusion criteria* encompassed all medical students registered in the School of Medicine at the University of Nottingham. Students were encouraged to engage in both survey and semi-structured interview processes regardless of their academic standing in pre-clinical and clinical years. Participants were selected using a convenience sampling technique, covering medical students aged between 18 and 41 who were registered in the School of Medicine. No *exclusion criteria* were applied. Participation was voluntary, with informed consent obtained prior to involvement. Given the sensitive nature of the research and the potential for disclosures regarding mental health issues and self-harm, strict ethical considerations were observed. Information on accessing student welfare services and helpline support services was provided. This study has received Research Ethics Committee approval from the University of Nottingham Faculty of Medicine and Health Sciences [ref no: FMHS 1561-1120]. The study adhered rigorously to the research and ethics conduct of the University. The information gathered from both the questionnaire and interviews underwent pseudonymisation to safeguard student identities, enabling withdrawal of consent if desired. Storage of this anonymised data occurred within a secure online repository accessible exclusively to investigators.

### Procedure

Following ethical approval, a study flyer was disseminated to invite medical students to participate. The distribution utilised various communication channels, including common areas within the school of medicine and university-wide online platforms like Facebook and Twitter. Adhering to safety protocols during the COVID-19 pandemic, the study flyer was electronically distributed rather than physically posted on noticeboards. All quantitative and qualitative data was collected between the period of 11 March 2021 and 30 March 2023, and the data was accessed for analysis on 1^st^ April 2023. Interested participants could directly reach the survey by following the link on the flyer, guiding them to the online questionnaire platform (JISC Online Surveys). A participant information sheet with detailed study information was available on the JISC online platform. Prior to questionnaire completion, all participants provided written informed consent.

The questionnaire’s consent form contained an extra question, offering participants interested in the interview part to share their email addresses and consent to being invited to participate to a subsequent semi-structured interview. Upon receiving their consent, the researcher reached out to the participants upon completion of the questionnaire. Following this initial contact, the researcher provided the participant information sheet and consent form for the qualitative phase of the study. Subsequently, after obtaining written informed consent, interview dates and times were scheduled based on participants’ availability, all conducted on Microsoft Teams. Following the completion of the questionnaires, a prize draw took place, allowing one participant the opportunity to secure a £50 Amazon gift voucher. Additionally, one participant from the interview part was selected to receive a £50 Amazon gift voucher through the draw.

### Measures-quantitative part

Demographic information was collected on age, gender and year of study (see Table 1).

**Table 1:** Demographic information.

Demographics	N	Percentage
Age		
18–21	111	43.0%
22–25	106	41.1%
26–29	35	13.6%
30–33	4	1.6%
34–37	1	0.4%
38–41	1	0.4%
Gender		
Female	163	63.2%
Male	95	36.8%
Year of study		
Pre-clinical	122	47.3%
Clinical	136	52%

#### Hospital anxiety and depression score.

The Hospital Anxiety and Depression Scale (HADS) [40] is a widely used self-report instrument for assessing anxiety and depression. It comprises 14 items, with two subscales, each scoring from 0 to 21. Scores of 8–10 indicate borderline anxiety/depression, and scores of 11–21 indicate severe anxiety/depression. HADS exhibits robust psychometric properties, as evidenced by a specificity of 0.78 and sensitivity of 0.9 for anxiety, along with a specificity of 0.79 and sensitivity of 0.83 for depression [41,42]. It has been widely utilised in various populations, including members of the general population [43] and medical students [44,45] in the UK.

#### Perceived stress scale (PSS-10).

The PSS-10 [46] is a 10-item questionnaire that assesses an individual’s perceived stress in the past month. Respondents indicate the frequency of their thoughts and feelings on a scale from 0 (never) to 4 (very often). It uses a Likert scale of 5 points, and the scores of the four positive statements (items 4, 5, 7, 8) are reversed. The total score spans from 0 (indicating no stress) to 40 (indicating high stress), with higher scores suggesting greater stress levels. The PSS-10 demonstrates strong internal consistency, as reflected in a Cronbach alpha of 0.70 [47].

#### The satisfaction with life scale (SWLS).

The SWLS [48] is a self-report questionnaire used to assess individuals’ general contentment with their lives. The survey comprises five questions, each item being evaluated on a 7-point Likert scale ranging from “strongly disagree” to “strongly agree.” The composite score, which ranges from 5 to 35, reflects satisfaction levels, with higher values denoting higher levels of satisfaction. The scale’s reliability is confirmed with a Cronbach’s alpha of 0.87 [48].

#### Personality traits (Big five inventory-BFI).

The 44-item “Big Five” Inventory [49] analyses personality traits using the Five-factor model. It employs a 5-point Likert scale, ranging from “strongly disagree” to “strongly agree”, including reverse-scored items 6, 2, 8, 9, 12, 18, 21, 23, 24, 27, 31, 34, 35, 37, 41 and 43. The BFI assesses extraversion (sociable, active, assertive, adventurous, outgoing and positive emotions), conscientiousness (organisation, careful behaviour, persistent, competence, self-discipline and achievement-oriented), agreeableness (trust, straightforward, cooperation, warmth, sympathy and modesty), and neuroticism (anxiety, deprecation shy, impulsivity, vulnerability and hostility), and openness (imaginativeness, curiosity, sensitivity, unconventional, artistic and a need for variety). Personality trait scores are calculated by averaging or summing responses to relevant items in each trait category. These scores reveal the strength or presence of each personality trait. The BFI-44 scales have demonstrated high levels of reliability (0.85) and validity (0.63) across different cultures [49] and are widely used for personality assessment.

#### Brief cope inventory-(COPE).

The Brief-COPE [50] consists of 28 items on a 4-point scale, with 1 representing “I do not do it at all” and 4 representing “I do it very much”, with alternatives from 0 (“I never do this”) to 3 (“I always do this”). The 28 items are divided into 14 coping subscales, which are then grouped under three main coping strategies: problem-focused, emotion-focused, and avoidant-focused coping. Individuals scoring high on the problem-focused subscale tend to utilise practical strategies to tackle and resolve challenges. In contrast, high or low scores on the emotion-focused subscale might not necessarily correspond directly with either psychological distress or wellbeing. However, lower scores on the avoidant subscale typically indicate a higher potential for adaptive coping. The internal consistency of the measure, as assessed by Cronbach’s Alpha (α), is reported to be 0.60 [50].

### Qualitative part

The research team developed the semi-structured interview guide after a comprehensive literature review and collaboration with academic staff and students. One pilot interview was conducted to improve the flow and depth of the interview by effectively using prompts. Each interview, lasting between 20–60 minutes, was audio-recorded and transcribed verbatim. The initial 5 minutes were dedicated to building rapport between the researcher and participants to ensure their comfort. The interview structure began with open-ended questions like “What influenced your decision to pursue medicine?”. It then transitioned to probing questions, for example, “How have you attempted to cope with stressful situations? “. The guide incorporated further probes to encourage more detailed or in-depth responses. The questions explored the medical students’ perceptions of the positive and negative factors that influence their psychological wellbeing, how current stressors impact their wellbeing, and the type of stressors they encounter before and during COVID-19. Additionally, the questions focused on how students manage stressors, including coping strategies.

### Data analysis

In the quantitative phase, the raw questionnaire data was organised and entered into SPSS (version 27). Descriptive statistics such as mean, frequency, percentage, and standard deviation were utilised to interpret the dataset. Furthermore, normality tests were conducted alongside Pearson’s correlation to examine the relationship and assess the strength of the correlation among the variables. Moreover, the data underwent screening to identify any missing values and outliers. Additionally, this study explored the impact of coping strategies (problem-focused, emotion-focused, avoidance) on psychological wellbeing outcomes (stress, depression, anxiety, life satisfaction) using Generalized Linear Regression Models (GLMs), with separate models tailored for each psychological wellbeing outcomes. Furthermore, an investigation into the influence of personality traits (extraversion, agreeableness, conscientiousness, neuroticism, openness) on coping strategies was conducted through GLMs. To ensure comparability across strategies, all values were standardised to z-scores.

The study further conducted additional GLMs to examine how personality types might have modulated the effectiveness of coping strategies in psychological wellbeing outcomes by incorporating interactions between coping strategies and personality types. The analysis included the main effects of the coping strategies and personality types, as well as interactions between each coping strategy and its corresponding personality type, resulting in 15 interaction variables.

Lastly, the study employed mediation analysis techniques using PROCESS (Version 4.1) to investigate whether problem-focused coping acts as a mediator in the relationship between stress and depression.

For the qualitative data obtained through semi-structured interviews, thematic analysis was conducted using Braun & Clark’s well-established six-step methodology [39]. This approach facilitated the identification of patterns and themes within the qualitative data. The themes were formulated based on the rich content of the data, allowing for an inductive thematic analysis approach to identify previously unreported themes. The semi-structured interviews were conducted by AH and transcribed by both AH and ES, with ES transcribing 70% of the material. AH and ES recorded initial thoughts to familiarise themselves with the data before creating preliminary codes, which were discussed with the research team, including EN and ES. One medical student reviewed their interview transcription, and AH incorporated all the suggested corrections. The codes were identified throughout the entire dataset using NVivo software (Version 12), and a list of codes was produced and examined until a consensus was reached. As the study progressed, a streamlined process was adopted, focusing on core themes to enhance the clarity and coherence of the research outcomes.

## Results

### Overview – Quantitative part

This section provides an in-depth analysis of the results obtained in the quantitative phase. The study involved the administration of five questionnaires to medical students, which measured various aspects, including stress, anxiety, depression, life satisfaction, personality traits, and coping strategies. The raw data was extracted from JISC online surveys and then transferred to SPSS for score computation and analysis. The dataset was thoroughly screened to identify and address any missing values. Additionally, reverse scoring was applied to specific measures, such as the Perceived Stress Scale (PSS) and the Big Five Inventory (BFI), to ensure consistency in the data. A total of n = 258 participants completed the survey over the course of several recruitment waves (n = 4), which commenced in March 2021 and concluded by March 2023. Among these respondents, 63.2% were female medical students (n = 163), and 36.8% were male medical students (n = 95), as shown in Table 1. In parallel with the quantitative phase, n = 25 medical students participated in interviews to provide qualitative insights, as illustrated in Table 11. This mixed-methods approach allowed for an examination of the factors influencing the psychological wellbeing of medical students.

#### Descriptive statistics.

Table 2 below presents descriptive statistics for the set of questionnaires administered to 258 medical students. The table includes information on the mean, standard deviation, skewness, and kurtosis.

**Table 2:** Descriptive statistics on the quantitative measures.

Questionnaire	Mean	St. Deviation	Skewness	Kurtosis
Satisfaction with life scale	19.17	8.093	.084	−1.119
Perceived stress scale	21.96	6.821	−1.193	.144
Hospital anxiety and stress scales				
Anxiety	11.62	4.726	−.169	−.530
Depression	8.68	5.125	.056	−.707
The brief cope				
Problem-focused	21.45	5.012	−.312	.237
Emotion-focused	30.03	5.763	−.349	.648
Avoidance	17.49	4.848	.028	−.810
Big five personality				
Openness	32.62	2.611	−.787	.601
Neuroticism	23.18	3.309	−.545	.422
Conscientiousness	29.41	3.137	.529	−.762
Extraversion	25.66	3.999	.589	−.151
Agreeableness	29.18	3.546	.006	−.591

#### Normality of data.

The distribution of the data was assessed using the Kolmogorov-Smirnov test, primarily chosen due to the robustness of this test when dealing with a substantial sample size (n = 258). While the initial impression might suggest that the data deviate from a normal distribution, upon closer examination of the tested variables and a detailed review of the q-q plots and histograms (see S1 File), one can discern grounds to consider that the data collected from the sample exhibit a reasonably close approximation to a normal distribution. The departure from normality assumptions becomes less significant, particularly when sample sizes exceed 30 or 40 [51]; thus enabling the adoption of parametric analytical methods [52].

#### Correlations among stress, depression, anxiety, life satisfaction, coping strategies and personality traits.

Medical students reported a slightly low level of life satisfaction (M = 19.17, SD = 8.093), moderate to high levels of stress (M = 21.96, SD = 6.821), borderline to high levels of depression (M = 8.68, SD = 5.125) and high levels of anxiety (11.62, SD = 4.726). The predominant coping strategy among students was found to be emotion-focused (M = 30.03, SD = 5.763), followed by problem-focused coping (M = 21.35, SD = 5.012). Conversely, avoidance coping strategies were the least utilised among the students (M = 17.49, SD = 4.848). The personality profile of medical students showed higher mean scores in openness (32.62, SD = 2.611), followed by conscientiousness (29.4, SD = 3.137) and agreeableness (29.18, SD = 3.456), as compared to the mean score of extraversion (25.66, SD = 3.999) and neuroticism (23.18, SD = 3.309).

In order to illustrate the complex interplay among different aspects, Pearson’s correlations were employed to assess the relationships among several variables. These encompassed depression, anxiety, stress, life satisfaction, coping strategies (encompassing problem-focused coping, emotion-focused coping, and avoidance), and personality traits (extraversion, agreeableness, conscientiousness, neuroticism, and openness). The correlation coefficients are presented in Table 3. Generally, the results indicated correlations among the outcomes of depression, anxiety, stress, and life satisfaction. Specifically, life satisfaction was negatively correlated with perceived stress, depression, and anxiety. Anxiety and depression were positively correlated, as were anxiety and stress; and depression and stress.

**Table 3:** Correlation analyses among all quantitative variables.

	Variables	1	2	3	4	5	6	7	8	9	10	11	12
1	Satisfaction With Life Scale	1	−.588**	−.679**	−.607**	.338**	−0.042	−.448**	0.047	−0.012	−0.03	0.007	0.007
2	Perceived Stress Scale	−.588**	1	.691**	.717**	−.341**	0.037	.379**	−0.013	0.002	0.033	−0.058	−.132*
3	Depression	−.679**	.691**	1	.760**	−.377**	0.028	.576**	−0.047	0.06	0.07	−0.085	−0.079
4	Anxiety	−.607**	.717**	.760**	1	−.201**	.129 *	.464**	−0.004	0.018	0.035	−0.008	−0.106
5	PF Coping	.338**	−.341**	−.377**	−.201**	1	.657**	0.002	0.071	−0.091	−0.089	0.004	−0.067
6	EF Coping	−0.042	0.037	0.028	.129 *	.657**	1	.446**	0.088	−0.019	−.140*	−0.041	−.139*
7	Avoidance Coping	−.448**	.379**	.576**	.464**	0.002	.446**	1	0.007	0.037	−0.026	−0.065	−0.084
8	Openness	0.047	−0.013	−0.047	−0.004	0.071	0.088	0.007	1	0.085	−.453**	.537**	0.122
9	Neuroticism	−0.012	0.002	0.06	0.018	−0.091	−0.019	0.037	0.085	1	−.146*	0.064	.215**
10	Conscientiousness	−0.03	0.033	0.07	0.035	−0.089	−.140*	−0.026	−.453**	−.146*	1	−0.046	.333**
11	Extraversion	0.007	−0.058	−0.085	−0.008	0.004	−0.041	−0.065	.537**	0.064	−0.046	1	.489**
12	Agreeableness	0.007	−.132*	−0.079	−0.106	−0.067	−.139*	−0.084	0.122	.215**	.333**	.489**	1

Note: PF coping, problem-focused coping; EF coping, emotion-focused coping; statistical significance is indicated as p < 0.05* , p < 0.01** (2 – tailed).

The psychological wellbeing outcomes showed correlations with coping strategies. Problem-focused coping was linked positively to life satisfaction and negatively to depression, anxiety, and stress. Avoidant-focused coping correlated positively with depression, anxiety, and stress, and negatively with life satisfaction. Emotion-focused coping solely correlated positively with anxiety, while also showing positive correlations with conscientiousness and agreeableness. Stress demonstrated a negative correlation with agreeableness.

#### Effect of coping strategies on psychological wellbeing outcomes (stress, depression, anxiety, life satisfaction) using GLMs.

The analysis yielded multiple findings about the impact of predictor variables on participants’ stress, depression, anxiety, and life satisfaction.

***Main effects of the predictor variables of coping strategies on life satisfaction*:** The analysis revealed several noteworthy results regarding the effects of predictor variables, namely coping strategies, on participants’ life satisfaction (refer to Table 4). A regression analysis revealed a significant relationship [F(3, 254) = 41.075, p < 0.001], yielding an R-squared value of 0.327. This R-squared value signifies that the predictor variables can collectively explain approximately 32.7% of the variance in participants’ satisfaction with life. Specifically, the results revealed the following:

**Table 4:** Results of the generalized linear regression model with the outcome variable being life satisfaction.

Dependent variable: Satisfaction With Life Scale						
	Standardised coefficients B	Standard errors	F-values	P-values	Confidence interval	
Problem-focused coping	.453	.076	35.844	.001	.304	.602
Emotion-focused coping	−.173	.084	4.212	.041	−.340	−.007
Avoidance coping	−.372	.064	33.999	.001	−.497	−.246

Problem-focused coping: The data positively influenced satisfaction with life (β = 0.453, p < 0.001). The avoidant-focused coping predictor negatively influenced life satisfaction (β = −0.372, p < 0.001). Additionally, the analysis showed a negative relationship between emotion-focused coping and satisfaction with life (β = −0.173, p = 0.041).

***Main effects of predictor variables (coping strategies) on perceived stress:*** A regression analysis revealed a significant relationship [F(3, 254) = 33.427, p < 0.001], with an R-squared value of 0.283. This R-squared value indicates that the examined predictor variables can explain approximately 28.3% of the variance in participants’ perceived stress, as noted in Table 5. Specifically, the results showed the following:

**Table 5:** Results of the generalized linear regression model with the outcome variable being perceived stress.

Dependent variable: Stress						
	Standardised B	Standard errors	F-values	P-values	Confidence interval	
Problem-focused coping	−.504	.053	30.217	.001	−.657	−.350
Emotion-focused coping	.247	.087	8.056	.005	.076	.419
Avoidance coping	−270	.066	16.861	.001	.140	.399

Problem-focused coping: These data revealed a significant negative influence on perceived stress (β = −0.504, p < 0.001). Avoidant-focused coping was associated with increased perceived stress (β = 0.270, p < 0.001), and the analysis indicated a significant positive relationship between emotion-focused coping and perceived stress (β = 0.247, p = 0.005).

***Main effects of the predictor variables of coping strategies on depression and anxiety:*** The analysis revealed significant relationships for both anxiety [F(3, 254) = 30.437, p < 0.001] and depression [F(3, 254) = 76.754, p < 0.001], with R-squared values of 0.264 and 0.475, respectively. These values indicate that approximately 26.4% of the variance in participants’ anxiety and 47.5% of the variance in depression scores can be explained by the examined predictor variables.

Specifically, for depression, problem-focused coping showed a significant negative influence (β = −0.411, p < 0.001), indicating lower depression scores among individuals using this coping strategy. Conversely, avoidant-focused coping had a significant positive impact (β = 0.554, p < 0.001), while emotion-focused coping showed no significant impact (β = 0.051, p = 0.495).

Regarding anxiety, problem-focused coping demonstrated a significant negative influence (β = −0.298, p < 0.001), suggesting lower anxiety scores for individuals employing this strategy. Conversely, avoidant-focused coping had a significant positive impact (β = 0.400, p < 0.001) on anxiety scores, while emotion-focused coping had a borderline significant impact (β = 0.146, p = 0.023), as illustrated in Table 6.

**Table 6:** Results of the generalized linear regression model with the outcome variables being depression and anxiety.

Dependent variable: Depression						
	Standardised B	Standard errors	F-values	P-values	Confidence Interval	
Problem-focused coping	−.411	.067	37.998	.001	−.543	−.280
Emotion-focused coping	.051	.075	.468	.495	−.096	.198
Avoidance coping	.554	.056	97.075	.001	.443	.665
Dependent variable: Anxiety						
	Standardised B	Standard errors	F-values	P-values	Confidence Interval	
Problem-focused coping	−.298	.054	14.194	.001	−.106	.106
Emotion-focused coping	.129	.088	5.748	.038	−.028	.320
Avoidance coping	.400	.067	36.057	.001	.269	.531

#### Exploring the influence of personality traits on coping strategies.

GLMs were employed to investigate the influence of personality traits on coping strategies. The analysis revealed no statistically significant relationships between most personality traits and the coping strategies adopted by participants. However, exceptions were noted for conscientiousness and agreeableness. Both conscientiousness (β = −0.140, F(1,256) = 5.124, p < .024) and agreeableness (β = −0.139, F(1,256) = 5.040, p < .026) exhibited significant negative predictions for emotion-focused coping. The value of R Square showed that approximately 20% of the variance in emotion-focused coping can be attributed to the examined predictor variables, as noted in Table 7.

**Table 7:** Results of the generalized linear regression model with the outcome variable being coping strategies.

Dependent variable: Emotion-focused coping						
	Standardised B	Standard errors	F-values	P-values	Confidence Interval	
Conscientiousness	−.140	.011	5.124	.024	−.045	−.003
Agreeableness	−.139	.011	5.04	.026	−.045	−.003
Openness	.545	.492	1.231	.268	−.423	1.514
Extraversion	−.243	.493	.243	.623	−1.214	.728
Neuroticism	−.064	.379	.028	.867	−.809	.682

#### Impact of personality traits on the effectiveness of various coping strategies in psychological wellbeing outcomes (depression, anxiety, stress, life satisfaction).

The analysis revealed that personality traits have only had impact on coping strategies with depression and anxiety. A combination of certain coping strategies and personality traits influenced depression levels. Specifically, the interaction between problem-focused*agreeableness, emotion-focused*agreeableness, emotion-focused*conscientiousness and avoidance*extraversion exerted more substantial impact on depression than their individual contributions. The presence of agreeableness traits enhanced the effectiveness of problem-focused coping in reducing depression levels by a magnitude of −0.266 (p < 0.001). Moreover, the presence of agreeableness traits also strengthened the effectiveness of emotion-focused coping by 0.223 (p < 0.002). Similarly, the presence of conscientiousness traits supported the impact of emotion-focused coping on depression by −0.105 (p < 0.043), while the presence of extraversion traits enhanced the effectiveness of avoidance coping by −0.101 (p < 0.038). This interaction explains 3% of the variation in depression, corresponding to an increase in the R-squared of the model without interaction from 47% to 50% for the model with interaction(refer to Table 8).

**Table 8:** Results of the generalized linear regression model with interaction on the impact of personality traits on the effectiveness of various coping strategies for depression.

Dependent variable: Depression				
	Coefficient B	Standard errors	F-values	P-values
Problem-focused	−.383	.065	34.470	.001
Emotion-focused	.082	.074	1.222	.270
Avoidance	.535	.055	95.317	.001
Agreeableness	−.083	.059	2.004	.158
Extraversion	−.028	.061	.209	.648
Conscientiousness	.135	.057	5.642	.018
Neuroticism	.035	.047	.541	.463
Openness	.068	.061	1.266	.262
Problem-focused coping*Agreeableness	−.266	.063	17.973	<.000
Emotion-focused coping*Agreeableness	.223	.071	9.892	.002
Emotion-focused coping*Conscientiousness	−.105	.052	4.138	.043
Avoidance coping*Extraversion	−.101	.049	4.354	.038

Additionally, the analysis uncovered an interplay between coping strategies and personality traits that influenced anxiety levels. In particular, these interactions showed that problem-focused * agreeableness, emotion focused*agreeableness and avoidance*extraversion exerted more impact on anxiety than their individual contributions. Specifically, the presence of agreeableness traits amplified the effectiveness of problem-focused coping, resulting in a noteworthy reduction of anxiety levels by −0.254 (p < 0.001). Notably, this amplification effect extended to emotion-focused coping, with agreeableness traits boosting its efficacy by 0.266 (p < 0.001). Meanwhile, extraversion traits enhanced the effectiveness of avoidance coping by −0.160 (p < 0.005). These interactions collectively explain 5% of the variation in anxiety levels; the model’s R-squared value increased from 25% to 30% when incorporating these interactions, underscoring their significant role in the overall predictive capacity of the model, as shown by the data in Table 9.

**Table 9:** Results of the generalized linear regression model with interaction on the impact of personality traits on the effectiveness of various coping strategies for anxiety.

Dependent variable: Anxiety				
	Coefficient B	Standard errors	F-values	P-values
Problem-focused	−.304	.078	15.374	.001
Emotion-focused	.156	.086	3.260	.072
Avoidance	.377	.065	33.709	.001
Agreeableness	−.156	.069	5.061	.025
Extraversion	.081	.073	1.256	.264
Conscientiousness	.138	.068	4.167	.042
Neuroticism	.005	.056	.008	.927
Openness	.030	.072	.178	.673
Problem-focused coping*Agreeableness	−.254	.075	11.579	.001
Emotion-focused coping*Agreeableness	.266	.078	11.503	.001
Avoidance coping*Extraversion	−.160	.057	7.896	.005

#### The role of problem-focused coping as a mediator in the relationship between stress and depression.

Mediation analyses were performed to examine the mediation effects of Problem-Focused (PF) coping on the relationship between stress and depression in the University of Nottingham medical students (n = 258).

**Fig 1:**
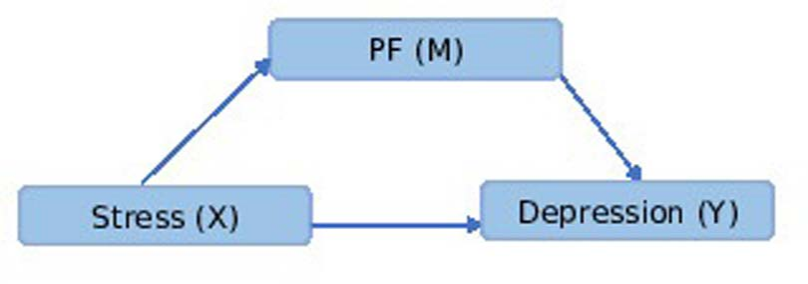
Diagram illustrating the mediation effect pathway.

Mediation analysis results showed that the total effect of stress (x) on depression (y), ignoring the mediator, was positively significant (B = .0691, se = 0.045, t = 15.312, p < 0.000). Even when accounting for PF coping, stress still exerted a significant direct influence on depression (B = 0.637, se = 0.047, t = 13.533, p < 0.000). Furthermore, the mediation analysis indicated a significant indirect effect of stress on depression through PF coping (B = 0.054; 95% CI = 0.021, 0.093), as shown in Table 10. These findings collectively suggest that PF coping plays a partial mediating role in the stress-depression relationship among medical students, as depicted in Fig 1.

**Table 10:** The mediation effects of PF (M) in the relationship between stress (X) and depression (Y).

************** TOTAL, DIRECT, AND INDIRECT EFFECTS OF X ON Y **************							
Total effect of Stress on Depression							
	Effect	se	t	p	LLCI	ULCI	c_cs
	6914	.0452	15.3120	.0000	.6025	.7803	.6914
Direct effect of X on Y							
	Effect	se	t	p	LLCI	ULCI	c’_cs
	6370	.0471	13.5336	.0000	.5443	.7297	.6370
Indirect effect(s) of X on Y:							
	Effect	BootSE	BootLLCI	BootULCI			
PF	.0544	.0183	.0212	.0932			

**Table 11:** Demographic characteristics for the subset of participants (n = 25) who took part in the qualitative study.

Demographics of Participants	Number (N, %)
Gender	
Female	21(84%)
Male	4 (16%)
Age In Years	
18–21	12 (48%)
22–25	9 (36%)
26–29	2 (8%)
30–33	2 (8%)
34–37	0 (0%)
38–41	0 (0%)
Year In School	
Pre-Clinical	5 (20%)
Clinical	20 (80%)
Undergraduate	22 (88%)
Graduate-Entry Students	3 (12%)

### Qualitative results

#### Overview.

Qualitative data were collected from a subset (n = 25) of the participants who took part in the quantitative study. The demographic compositions of these groups can be seen in Table 11.

#### Thematic analysis results.

During the thematic coding stage, the initial data were coded and grouped with headers, with20 topics emerging as groups became more diverse. The topics were re-organised and aggregated to identify the most predominant codes relevant to the research questions. Early classifications were refined into advanced concepts or themes, while some themes were merged into others. Sub-themes were used to focus on particular elements within the theme. Some sub-themes were removed as they lacked enough views from participants, such as “Academic and professional uncertainty.” After iterative discussions and reviews, recurring themes emerged throughout the entire data, and the researchers reached a point of data saturation. A reflexive log was maintained to record and analyse data transcriptions, coding, personal reflections, or observations that came up throughout the analysis process. These findings were further discussed with EN and ES for additional insight and clarity, ensuring transparency in the methodology. The final themes consisted of 3 distinct feature categories. Within these overarching categories, 5 themes were generated in total, with respective subthemes, i.e., two themes on ‘factors adversely impacting medical students’ psychological wellbeing’; two themes on ‘factors positively impacting medical students’ psychological wellbeing’; and one theme on ‘coping strategies used by medical students to manage their psychological wellbeing’.

***Category 1: Factors adversely impacting medical students’ psychological wellbeing.***

Codes were grouped according to key factors adversely affecting medical students’ psychological wellbeing, including academic, personal and organisational issues. Additionally, students discussed the impact of COVID-19 on their academic, personal, and social lives. Detailed descriptions of themes 1–2 and their corresponding subthemes can be found in S2 File. Representative quotes from participants’ respective transcripts have been included in S3 File.

**Theme 1 - Perceived adverse academic, personal and organisational factors contribute to poor wellbeing**

The term poor wellbeing was attributed when students spoke about stress-related academic, personal and organisational factors. When talking about poor wellbeing, students were able to articulate their feelings perceived to be associated with poor wellbeing and were able to identify academic (subthemes 1 a,b,c), personal (subtheme 1 d,e), and organisational (subthemes 1 d) factors that contribute to poor wellbeing.

***Subtheme 1a - Excessive workloads and exam concerns were sources of stress and low mood*.**

Many students described a large amount of content as ‘insane’ and the overwhelming burden of knowledge needed to study for exams as stressful. Furthermore, they claimed that the course requires studying broadly and deeply simultaneously, and that there is never enough time to study or finish studying. **Quote 1, P11F, Year 3**

Many also felt quite stressed with exams coming up, and having first-year exams counting toward final-year ranking was highlighted as particularly stressful due to the possible negative impact on their life. Students claimed that by having the first exams count toward final year grades, they would study just as hard in their first year as they would in later years, and they would not build a foundation of life apart from medicine. **Quote 2, P13, F, Year 4**

Alongside the pressure of the content and the exams, students believed that their courses were stressful compared to other courses, as the pressure of the medical course was present throughout the degree; unlike, as reported, in other courses wherein students have more free time outside their degree. **Quote 3, P8, F, Year 3**

***Subtheme 1b - Adjusting to a new environment was challenging, stressful and emotionally heavy*.**

Periods of transition, such as first starting medical school and first starting placement, were perceived to be very stressful. Students reported finding themselves in new, unfamiliar environments with more expectations imposed on them. Additionally, many students described feeling ‘useless’ and ‘uncomfortable’. Other students found exposure to sick and suffering people regularly stressful because it distorted their worldview assumption that everyone is unhappy. **Quote 4, P8, F, Year 3**

***Subtheme 1c - Students experience a lack of study-life balance due to increased workloads*.**

A large subset of students expressed concerns about time commitments and study-life balance. For example, the workload involved in clinical training in medicine, which requires managing time between academic work (teaching, studying for exams) and long hours in placement, was termed as ‘unrealistic’. This relentless balancing act often led to feelings of exhaustion, reduced energy levels, and a persistent challenge in task prioritization. **Quote 5, P1, F, Year 3**

Students also reported struggling to balance personal and academic work and noted that they sacrificed their personal lives. The majority of students stated that medicine had become the only domain preoccupying their lives

As a consequence of this imbalance in their lives, many students felt that their interpersonal connections had become strained. **Quote 6, P24, F, Year 4**

For some students, medical school had an effect on their good mood. **Quote 7, P15, M, Year 4**

***Subtheme 1d - The medical school learning environment (culture) and students’ perceptions about self and the course were understood to be sources of stress*.**

Medical students believed that the medical school course is their baseline stress*,* with the learning environment imposing pressure on them to compete.

Students felt pressurised having to work harder, put in extra effort and do all the extracurricular activities as well. **Quote 8, P1, F, Year 3**

In addition, the culture of medical school was perceived to set a high standard, making them often believe they needed to be better. As a result, many students reported trying to create images of perfection while feeling incompetent and like an imposter. **Quote 9, P8, F, Year 3**

***Subtheme 1e - Having a mental health disorder and financial concerns added an additional burden on medical students’ ability to manage a stressful course*.**

Students reported experiencing mental health challenges such as anxiety and depression, which seemed to complicate their social and academic lives, alongside financial concerns. **Quote 10, P10, F, Year 4**

**Theme 2 - During COVID-19, medical students were impacted negatively at an academic, personal and social level**

Medical students commented on facing additional challenges to their medical education studies presented by the pandemic, which adversely affected their academic (subtheme 2a,b), personal (subtheme 2c) and social (subtheme 2d) lives.

***Subtheme 2a - Lack of communication with medical school during COVID-19 caused significant distress.***

Students shared concerns about the medical school’s communication regarding the restructuring of course schedules and assessments, feeling it could have been handled more effectively; they also noted that support during the transition was limited, particularly before exams, with the cancellation of mock exams due to COVID-19 causing notable distress. **Quote 11, P25, M, Year 4**

***Subtheme 2b – COVID-19 decreased the amount of clinical training, and students felt more stressed and less prepared as a doctor in training*.**

Students also discussed how COVID-19 had added additional pressure to deal with their already stressful course. Some students felt that their study-life balance had been even more skewed to the work side, putting more emphasis on studying, which was believed to have affected their study-life balance negatively. Students also expressed frustrations with limited placement opportunities and ward cancellations, which led to decreased clinical training (6th month), leading to feeling l’ess prepared as a doctor’. **Quote 12 P15, M, Year 4 & 12, P7, F, Year 4**

***Subtheme 2c – COVID-19 resulted in reduced coping potential and reduced motivations and social isolation*.**

Lack of motivation was commonly cited by medical students when asked about the impact of COVID-19. This was because most of the studying had moved to online modalities, and students felt they would find themselves in the same environment with the same people and found it hard to motivate themselves, thus becoming less productive. **Quote 13, P4, F, Year 5**

In addition to the decline in motivation, students noted challenges in coping strategies, and experienced difficulties using their usual support network, particularly during the peak of the pandemic. Many turned to physical activity as a means to alleviate stress and foster positive wellbeing. The closure of gyms due to COVID-19 health and safety regulations negatively impacted students’ physical fitness and exercise routines, making it challenging for them to manage stress. **Quote 14, P2, M, Year 5**

These struggles were further manifested in students’ responses to stressful situations, such as crowded places, where they reported to cautiously slow down, acknowledging the perceived uncontrollable nature of the situation**. Quote 15, P9, F, Year 2**

Furthermore, medical students noted the challenges of an accelerated curriculum in the first two years, emphasising that the stress and extensive learning were seen as controllable based on invested effort. However, the struggle with examination style varied in perceived controllability among students. Additionally, during COVID-19, reportedly uncontrollable factors such as limited socialization and remote learning led to feelings of loneliness, isolation, and negative impacts on student experience. **Quote 16, P20, F, Year 3 & Quote 19, P16, F, Year 3.**

***Category 2: Factors positively impacting medical students’ psychological wellbeing***.

Codes were grouped according to certain factors that positively impact medical students’ psychological wellbeing. This relates to aspects linked with the course of medicine and the reconciliation of study-life (theme 3). Another positive aspect identified is the beneficial changes students have noticed due to COVID-19 (theme 4). Detailed descriptions of themes 3–4 and their corresponding subthemes can be found in S4 File. Representative quotes from participants’ respective transcripts have been included in S5 File.

**Theme 3 – Certain aspects relevant to the medical school course and study-life balance were associated with a positive mood.**

This theme constitutes the positive views and experiences that medical students shared about their studies, including handling the work whilst having free time (subtheme 3a), having a sense of positive academic achievement (subtheme 3b), satisfaction from positive experiences during placement (subtheme 3c) and having quality time spent with close friends or family (subtheme 3d). These experiences were perceived as making medical students feel happy and well.

***Subtheme 3a - Managing work whilst also allowing for leisure time helped maintain a long-term positive perspective on (academic) life*.**

Many students acknowledged that achieving a study-life balance contributed positively to their sense of wellbeing. When students felt they could manage their time by having spare time to do things outside medicine as well as time for study, it helped promote a long-term optimistic outlook on their (academic) life. **Quote 17, P8, F, year 3**

***Subtheme 3b - A sense of achievement was reported as a good feeling that boosts the mood*.**

Similarly, receiving good results or being able to perform a medical procedure was reported to often improve student wellbeing. Although medical exams or dissertations were very demanding, students reported feeling a sense of pride and satisfaction after completion.

***Subtheme 3c - Direct contact with patients and professional support made medical students feel helpful and fulfilling*.**

While in the earlier subtheme placement experiences were believed to be a source of stress, they were also identified as the most influential aspect contributing positively to students’ wellbeing. The ability to put knowledge to use during placements gave them a sense of pride and purpose. They stated that interacting with patients, witnessing their recovery and experiencing something positive in the ward made them feel happy and helpful. Through hands-on experience and interaction with healthcare professionals, students felt they were able to appreciate the effort they put into learning. This first taste of clinical work seemed to allow students to learn substantial skills, boosting their attitude toward the career. They also viewed professional support as a source of motivation and inspiration. **Quote 18, P16, F, Year 3**

***Subtheme 3d - Meaningful relationships with friends and family and peer socialising brought about a sense of happiness*.**

Quality time spent with close friends, family or partners was perceived as essential to students’ happiness. Many students described a feeling of joy when they would have a supportive network. Being with peers who made them feel part of a community was believed to positively contribute to their wellbeing. **Quote 19, P5, F, Year 3**

**Theme 4 - COVID-19 had some positive impacts at an academic and personal level.**

Certain activities described by students during COVID-19 were thought to have had a beneficial influence, such as easy access to the online recorded lectures (subtheme 4a)and the ability to acquire new hobbies (subtheme 4b).

***Subtheme 4a - Online learning was found to be easier, with accessibility to the recorded lectures at any time*.**

While most participants felt demotivated by the transition to online teaching and study, some participants reflected on their ‘delightful’ (comfortable) experience, noting and appreciating the convenience of the recorded lectures. **Quote 20, P9, F, Year 2**

***Subtheme 4b - Developing new hobbies that could be sustained was deemed to be positive*.**

Many participants acknowledged the limited engagement in extracurricular activities during COVID-19; however, some developed new hobbies that were perceived to be helping them to relax and recharge, making them better able to handle the pressures of medical studies.

***Category 3: Coping strategies used to manage students’ psychological wellbeing***.

Medical students discussed the strategies they used to manage their psychological wellbeing. They seemed to have adopted active strategies to manage stress and mood (theme 5). Detailed descriptions of theme 5 and the corresponding subthemes can be found in S6 File. Representative quotes from participants’ respective transcripts have been included in S7 File.

**Theme 5 - Adopting active strategies to manage stress and mood.**

Medical students reported engaging in several activities that could be considered stress-relieving and uplifting.

***Subtheme 5a - Keeping an activity planner and a daily routine can help in managing stress*.**

When students felt overwhelmed, they resorted to a routine and schedule to achieve good time management and get through the day. Calendars and planners allowed them to set time limits for different activities and visualise the amount of time needed for specific tasks. This helped them feel in control and allowed easier goal achievement as everything was planned out.

***Subtheme 5b - Extracurricular activities provided a way of relaxation and helped maintain a positive mood*.**

The act of journalling reportedly allowed medical students to clarify and label their emotions, helped them better think through obstacles and reflect on different situations. The physicality and privacy of journalling made it a strong tool for those who enjoy using it. **Quote 21, P5, F, Year 3**

Some students mentioned that they would break up their day into various chunks to achieve more balance, for example by cooking or watching TV. This helped them rest and reset, leaving them more equipped to deal with the stress imposed on them as a medical student. **Quote 22, P13, F, Year 4**

***Subtheme 5c - Taking the time to reach out to others or to reflect on the stressful situation*.**

When faced with complex and challenging situations, some students approached problems practically and worked through them, making it helpful to focus on what they could do to manage their problems. **Quote 23, P2, M, Year 5**

Medical students articulated encounters with situations perceived as beyond their immediate control, necessitating adaptation to demanding schedules during placements. Simultaneously, they acknowledged the imperative to accept emotional challenges encountered during these experiences as integral to the learning process despite impacting their daily routines and occasionally inducing dissatisfaction. **Quote 24, P5, F, Year 3**

Additionally, they accepted stress as inevitable in their academic and professional journey, recognising certain aspects as uncontrollable. Rather than expecting a stress-free experience, they tended to seek moments of distraction to alleviate pressures.

## Discussion

The primary objective of this study was to explore the diverse positive and negative factors impacting medical students’ psychological wellbeing, coping strategies, and personality traits, utilising a mixed-methods approach. Quantitative data provided statistical insights into these aspects, while a qualitative analysis delved into stressors experienced by students, differentiating between pre-pandemic and pandemic periods, and their impact on psychological wellbeing; additionally, coping strategies were identified qualitatively. By merging findings from both qualitative and quantitative analyses, a more holistic comprehension of the research problem emerged; this approach is supported by the recognition that the combination of various research methods adds richness and comprehensiveness to a study [53,54].

Overall, the study showed a complex interplay among stress, life satisfaction, and the psychological wellbeing of medical students. The data indicated that these students commonly experience moderate to high levels of stress, supporting findings from earlier research [55,56]. This elevated stress level may, in turn, contribute to the slightly low level of life satisfaction reported by medical students. The pervasive stressors they face can take a toll on their overall wellbeing, resulting in reduced life satisfaction, as has been documented in the literature [13,57]. Furthermore, the data revealed moderate to high levels of depression and high levels of anxiety among medical students. These findings align with well-documented research showing that medical students are at a high risk of experiencing mental health issues, including anxiety and depression [9,58], emphasising the need for institutions to provide robust mental health support and interventions to address these concerns.

Understanding the inter-connectedness of psychological wellbeing outcomes, such as stress, depression, anxiety, and life satisfaction, is crucial for addressing medical students’ mental health; this study revealed that these aspects are intertwined and can influence each other in complex ways. High-stress levels can lead to decreased life satisfaction, increasing the risk of depression and anxiety. Conversely, students experiencing depression and anxiety may experience reduced life satisfaction. These findings are in agreement with existing literature [14,57] and further highlight the need for comprehensive mental health interventions that address the broader context of students’ psychological wellbeing, rather than focusing solely on individual symptoms.

While our study’s quantitative data offered insights into the general stress levels of medical students, the qualitative exploration through interviews delved into nuanced aspects of stress. This included the identification of academic stressors such as heavy workloads, exam concerns, time pressure, transitions, and conflicts in the study–life balance. Additionally, the interviews captured the personal and cultural dimensions of stress, encompassing mental health issues, financial concerns, and the impact of a competitive environment with high expectations and standards. These identified stressors align seamlessly with patterns identified in earlier literature [59,60].

Additionally, research suggests a link between academic demands and increased stress levels, correlating with decreased productivity and increased symptoms of depression and anxiety [61]. Our study confirmed these assumptions and revealed that prolonged exposure to stress can also negatively impact other aspects of students’ lives, including relationships with family and friends; students reported spending less time with loved ones and experiencing emotional strain, frustration, and difficulty in prioritising tasks, consistent with previous literature [9].

The global pandemic has compounded the challenges faced by medical students, intensifying existing stressors and introducing new obstacles to their educational journey, which resonates with previous research documenting the adverse effects of COVID-19 on mental health [62,63]. Our study indicated that medical students have reportedly increased levels of anxiety and depression, which is also consistent with previous research [62]. Additionally, dissatisfaction with online learning and feelings of unpreparedness for future careers compared to face-to-face teaching have been reported in our study, echoing earlier observations on the educational disruptions caused by the pandemic [63]. The disruptions in teaching schedules, elective placements, and travel restrictions seemed to have further strained students’ psychological wellbeing.

The current study employed an inclusive approach, actively seeking input from students to understand the factors influencing their wellbeing, delineating both negative and positive contributors. The existing literature has highlighted the critical importance of embracing a positive psychological framework when assessing the overall psychological wellbeing of individuals [64,65]. This framework not only ensures a thorough understanding of psychological health among medical students but also highlights factors that promote resilience, personal development, and overall positive functioning within this group. By incorporating a positive psychological perspective into this study, insights were gained into the challenges faced by medical students, as well as their strengths and resources for overcoming these challenges. This acknowledges that mental health is not just about the absence of illness as per the Dual Continuum Model of mental health and illness [85] but is also about the presence of positive emotions, engagement, relationships, meaning, and accomplishment, as outlined by Seligman [65]. Therefore, by focusing on elements that foster personal growth, resilience, and overall positive functioning, better support can be provided for the psychological wellbeing of medical students and help them succeed in their academic and professional pursuits. This approach can assist in identifying appropriate wellbeing support systems that both address negative aspects and enhance positive factors hence promoting a comprehensive approach to improving the mental health of medical students [66].

In this study, medical students identified positive aspects such as maintaining a study-life balance, positive academic achievements, fulfilling placement experiences, fostering relationships with staff and engaging in peer socializing. These findings align with earlier research pointing out the importance of positive contributors to students’ wellbeing [67].

Despite the perceived associated stress, placements and social connections seem to significantly mitigate challenges, with hands-on learning and supportive relationships fostering resilience and satisfaction, highlighting the value of experiential learning and social integration [20,67,68].

In terms of coping strategies, both qualitative and quantitative data revealed that coping strategies play a pivotal role in the psychological wellbeing of medical students. On a positive note, problem-focused coping seems to positively impact medical students’ psychological wellbeing, while emotion-focused and avoidance coping have less favourable effects. This pattern of findings aligns with findings from other studies [19,69] which observed a prevalent use of both problem-focused and emotion-focused coping, with avoidance coping being less frequently employed. Nevertheless, within the present dataset, the utilisation of emotion-focused coping did not emerge as a predictor for depression. This might suggest that relying on emotion-focused coping may exacerbate stress, anxiety and life satisfaction, but these aspects may not have the same impact on the development of depressive symptoms in the data. The relationship between emotion-focused coping and depression may be more complex and influenced by other factors, such as personality traits and external support systems [70,71].

The qualitative findings complemented these findings and showed that students mainly employed active coping strategies such as planning and problem-solving, as opposed to resorting to avoidant strategies such as alcohol and drug use, consistent with findings reported in previous studies [72].

Interestingly, the findings revealed a dual coping strategy among medical students, where a proactive, problem-solving orientation seems to be adopted in perceived controllable situations, fostering an increased sense of control and effectively alleviating stress and negative emotions. However, when faced with challenges beyond their immediate control, such as demanding schedules, placements, and the impact of COVID-19, their coping strategies shift towards accepting emotional challenges as an integral part of the learning process. This observation highlights the adaptive nature of medical students, showcasing a proactive stance in controllable situations and an emotionally resilient approach when confronting uncontrollable challenges.

Further, this study diverges from prior UK-based research [73–75] by revealing a lower prevalence of maladaptive coping strategies among medical students, potentially influenced by their advanced clinical years. Older clinical students tended to gravitate towards more adaptive coping methods, highlighting the role of age and experience [72,76,77]. Early support and a focus on proactive planning could bolster coping skills in younger students throughout their medical education.

Moreover, the findings reinforce the positive impact of physical and extracurricular activities on stress management for medical students, consistent with existing literature [78,79]. Engaging in activities like running or walking outside of medicine can significantly enhance mood and overall wellbeing, highlighting the importance of integrating such practices into stress management strategies for medical students.

Our data also highlighted that medical students’ personality profiles indicated higher scores in openness, conscientiousness, and agreeableness, which are associated with resilience in the medical field. Conversely, scores for extraversion and neuroticism were comparatively lower, consistent with previous research [80,81].

Moreover, this study explored how coping strategies and personality traits might affect depression and anxiety, finding that agreeableness and conscientiousness negatively predicted emotion-focused coping. These findings highlight the need for tailored interventions based on personality traits [82–84]. The study showed that extraversion moderates the impact of avoidance coping, with highly extraverted individuals experiencing less anxiety and depression than those with average extraversion, aligning with research on personality and coping strategies [18,25,84]. Furthermore, the study findings suggested problem-focused coping as a potential mediator between stress and depression, reducing depression risk and emphasizing the need for interventions to enhance problem-solving skills, as per prior research [22,82]. While psychological wellbeing outcomes were linked to coping strategies, regression models explained only moderate variability (e.g., 47.5% for depression), reflecting the complex interplay of such factors influencing mental health.

## Implications and future directions

This study’s findings highlighted key factors influencing medical students’ psychological wellbeing, offering guidance for mental health policies and interventions; they underscored the need for comprehensive strategies addressing medical students’ stress, depression, anxiety, and life satisfaction, while considering their personality traits and coping mechanisms. Tailored programs and holistic approaches are hence essential to mitigate high stress and mental health challenges in medical students.

Future research could expand on these findings by exploring diverse samples, including students from varied ethnic backgrounds and different educational settings. Longitudinal studies tracking changes in stress, depression, and coping strategies throughout medical education would provide deeper insights into the long-term impact of medical training and the effectiveness of support systems.

## Strengths and limitations

The study explored the intricate dynamics among stress, depression, anxiety, life satisfaction, coping strategies, and personality traits within the context of medical education. It examined the combined impact of various factors, including academic pressures, personal challenges, organisational support and social interactions, on students’ psychological wellbeing. One notable strength of the study lies in its significant contribution to the existing literature by employing a mixed methods approach and thoroughly examining both favourable and adverse influences, filling a critical gap in the literature. Traditionally, studies in this field have often focused primarily on negative aspects, overlooking the potentially important positive contributors to overall wellbeing. This study takes a more balanced approach by not only identifying challenges but also highlighting positive factors that foster psychological resilience among medical students. Additionally, the study examines how specific personality traits may shape individuals’ coping strategies and impact their mental health in the face of these varied stressors.

The findings hold real-world relevance for medical education and the healthcare sector, providing practical guidance for designing supportive learning environments and improving medical students’ mental health; these insights corroborate existing research on stress, anxiety, depression, life satisfaction, coping, and personality, further enriching the body of knowledge in this field.

It is important to underline the limitations of this study, including its restricted focus on a single medical school, the absence of data on the respondents’ ethnic and religious origins, and the small sample size which may restrict the generalizability of its findings. While the study is limited by its focus on a single medical school, it is important to note that this limitation may have minimal impact within a UK context where cultural factors are generally similar across different schools. However, it is crucial to acknowledge that cultural variations could potentially introduce different effects, especially in an international context. Therefore, conducting a more extensive study considering respondents’ ethnic and religious origins becomes essential to extrapolate these findings beyond a national scope. Additionally, the unique stressors of the COVID-19 pandemic may have influenced the results, underlining the importance of longitudinal studies to explore how such external factors impact psychological wellbeing over time.

## Conclusions

The present study suggested that it is essential to consider the impact of both positive and negative factors on the psychological wellbeing of medical students and to provide effective support on coping strategies to manage these factors. The research findings highlighted the numerous challenges that medical students face, which can have adverse effects on their mental health. Tailored support that considers individual personality traits is deemed critical in this context, as these traits were found to significantly impact coping mechanisms and mental health outcomes. The findings bear practical implications for medical institutions and educators, including the development of refined stress management programs and mental health support resources more generally, as well as curricula that promote effective problem-solving skills. These initiatives can promote the holistic development and resilience of future healthcare professionals.

## Supporting information

S1 File Histogram and Q-Q Plot for perceived stress scale, hospital anxiety depression scale, satisfaction with life scale, brief COPE and big five inventory. (DOCX)

S2 File Descriptions of themes 1–2 and their corresponding subthemes. (DOCX)

S3 File Category 1 quotes. (DOCX)

S4 File Descriptions of themes 3–4 and their corresponding subthemes. (DOCX)

S5 File Category 2 quotes. (DOCX)

S6 File Descriptions of theme 5 and the corresponding subthemes. (DOCX)

S7 File Category 3 quotes. (DOCX)

## References

[1] Cuttilan A, Sayampanathan A, Ho R-M. Mental health issues amongst medical students in Asia: a systematic review (2000–2015). Ann Transl Med. 2016;4(4). 10.3978/j.issn.2305-5839.2016.02.07 PMC4779785 27004219

[2] West M, Coia D. Caring for doctors caring for patients. General Medical Council. 2019.

[3] Dunn LB, Iglewicz A, Moutier C. A conceptual model of medical student well-being: promoting resilience and preventing burnout. Acad Psychiatry. 2008;32(1):44–53. doi: 10.1176/appi.ap.32.1.44 18270280

[4] Slavin SJ. Medical student mental health: culture, environment, and the need for change. JAMA. 2016;316(21):2195–6. doi: 10.1001/jama.2016.16396 27923076

[5] Yusoff MSB, Yee LY, Wei LH, Meng LH, Bin LX, Siong TC, et al. A study on stress, stressors and coping strategies among Malaysian medical students. Int J Stu Res. 2011;1(2):45–50. doi: 10.5549/ijsr.1.2.45-50

[6] Hill MR, Goicochea S, Merlo LJ. In their own words: stressors facing medical students in the millennial generation. Med Educ Online. 2018;23(1):1530558. doi: 10.1080/10872981.2018.1530558 30286698 PMC6179084

[7] Joshi S, Joshi C, Sayana A, Joshi AK. Impact of COVID 19 pandemic on the academics and psychology of final year medical students. Can Med Educ J. 2021;12(2):e110–1. doi: 10.36834/cmej.70528 33995730 PMC8105579

[8] Farrell SM, Moir F, Molodynski A, Bhugra D. Psychological wellbeing, burnout and substance use amongst medical students in New Zealand. Int Rev Psychiatry. 2019;31(7–8):630–6. doi: 10.1080/09540261.2019.1681204 31701792

[9] Dyrbye LN, Thomas MR, Shanafelt TD. Systematic review of depression, anxiety, and other indicators of psychological distress among U.S. and Canadian medical students. Acad Med. 2006;81(4):354–73. doi: 10.1097/00001888-200604000-00009 16565188

[10] Rotenstein LS, Ramos MA, Torre M, Segal JB, Peluso MJ, Guille C, et al. Prevalence of depression, depressive symptoms, and suicidal ideation among medical students: a systematic review and meta-analysis. JAMA. 2016;316(21):2214–36. doi: 10.1001/jama.2016.17324 27923088 PMC5613659

[11] Tempski P, Bellodi PL, Paro HBMS, Enns SC, Martins MA, Schraiber LB. What do medical students think about their quality of life? A qualitative study. BMC Med Educ. 2012;12:106. doi: 10.1186/1472-6920-12-106 23126332 PMC3527341

[12] Yusoff MSB, Abdul Rahim AF, Baba AA, Ismail SB, Mat Pa MN, Esa AR. The impact of medical education on psychological health of students: a cohort study. Psychol Health Med. 2013;18(4):420–30. doi: 10.1080/13548506.2012.740162 23140393

[13] Mineva K. Coping and life satisfaction relationship in medical students: the mediating role of perceived stress. JESP. 2022;12(1):129–37. doi: 10.51865/jesp.2022.1.13

[14] Kjeldstadli K, Tyssen R, Finset A, Hem E, Gude T, Gronvold NT, et al. Life satisfaction and resilience in medical school—a six-year longitudinal, nationwide and comparative study. BMC Med Educ. 2006;6:48. doi: 10.1186/1472-6920-6-48 16984638 PMC1592096

[15] Binswanger IA, Merrill JO, Krueger PM, White MC, Booth RE, Elmore JG. Gender differences in chronic medical, psychiatric, and substance-dependence disorders among jail inmates. Am J Public Health. 2010;100(3):476–82. doi: 10.2105/AJPH.2008.149591 19696388 PMC2820077

[16] Fares J, Al Tabosh H, Saadeddin Z, El Mouhayyar C, Aridi H. Stress, burnout and coping strategies in preclinical medical students. N Am J Med Sci. 2016;8(2):75–81. doi: 10.4103/1947-2714.177299 27042604 PMC4791902

[17] Connor-Smith JK, Flachsbart C. Relations between personality and coping: a meta-analysis. J Pers Soc Psychol. 2007;93(6):1080–107. doi: 10.1037/0022-3514.93.6.1080 18072856

[18] Smith-Han K, Martyn H, Barrett A, Nicholson H. That’s not what you expect to do as a doctor, you know, you don’t expect your patients to die. Death as a learning experience for undergraduate medical students. BMC Med Educ. 2016;16:108. doi: 10.1186/s12909-016-0631-3 27080014 PMC4832523

[19] Ramadianto AS, Kusumadewi I, Agiananda F, Raharjanti NW. Symptoms of depression and anxiety in Indonesian medical students: association with coping strategy and resilience. BMC Psychiatry. 2022;22(1):92. doi: 10.1186/s12888-022-03745-1 35130862 PMC8820032

[20] Jenkins TM, Kim J, Hu C, Hickernell JC, Watanaskul S, Yoon JD. Stressing the journey: using life stories to study medical student wellbeing. Adv Health Sci Educ Theory Pract. 2018;23(4):767–82. doi: 10.1007/s10459-018-9827-0 29730708

[21] Erschens R, Loda T, Herrmann-Werner A, Keifenheim KE, Stuber F, Nikendei C, et al. Behaviour-based functional and dysfunctional strategies of medical students to cope with burnout. Med Educ Online. 2018;23(1):1535738. doi: 10.1080/10872981.2018.1535738 30371222 PMC6211255

[22] Dodek P, Culjak A, Cheung E, Hubinette M, Holmes C, Schrewe B, et al. Active coping in medical students is associated with less burnout and higher resilience. C21 DETERMINANTS OF BURNOUT AND WELLNESS AMONG PHYSICIANS AND TRAINEES. American Thoracic Society; 2019. A4300 p.

[23] Steiner-Hofbauer V, Holzinger A. How to cope with the challenges of medical education? Stress, depression, and coping in undergraduate medical students. Acad Psychiatry. 2020;44(4):380–7. doi: 10.1007/s40596-020-01193-1 32080825 PMC7359127

[24] Park CL, Adler NE. Coping style as a predictor of health and well-being across the first year of medical school. Health Psychol. 2003;22(6):627–31. doi: 10.1037/0278-6133.22.6.627 14640860

[25] Afshar H, Roohafza HR, Keshteli AH, Mazaheri M, Feizi A, Adibi P. The association of personality traits and coping styles according to stress level. J Res Med Sci. 2015;20(4):353–8. doi: 10.4103/1735-1995.158255 26109990 PMC4468450

[26] Gramstad TO, Gjestad R, Haver B. Personality traits predict job stress, depression and anxiety among junior physicians. BMC Med Educ. 2013;13:150. doi: 10.1186/1472-6920-13-150 24207064 PMC3842670

[27] Lo C-L, Tseng H-T, Chen C-H. Does medical students’ personality traits influence their attitudes toward medical errors? Healthcare. 2018;6(3):101. 10.3390/healthcare6030101 PMC6163914 30126126

[28] Chen L, Qu L, Hong RY. Pathways linking the big five to psychological distress: exploring the mediating roles of stress mindset and coping flexibility. J Clin Med. 2022;11(9):2272. doi: 10.3390/jcm11092272 35566398 PMC9105170

[29] Hope V, Henderson M. Medical student depression, anxiety and distress outside North America: a systematic review. Med Educ. 2014;48(10):963–79. doi: 10.1111/medu.12512 25200017

[30] Almeida T, Kadhum M, Farrell SM, Ventriglio A, Molodynski A. A descriptive study of mental health and wellbeing among medical students in Portugal. Int Rev Psychiatry. 2019;31(7–8):574–8. doi: 10.1080/09540261.2019.1675283 31638442

[31] Yusoff MSB, Abdul Rahim AF, Yaacob MJ. Prevalence and sources of stress among universiti sains malaysia medical students. Malays J Med Sci. 2010;17(1):30–7. 22135523 PMC3216143

[32] Farrell SM, Kadhum M, Lewis T, Singh G, Penzenstadler L, Molodynski A. Wellbeing and burnout amongst medical students in England. Int Rev Psychiatry. 2019;31(7–8):579–83. doi: 10.1080/09540261.2019.1675960 31692396

[33] Farrell SM, Molodynski A, Cohen D, Grant AJ, Rees S, Wullshleger A, et al. Wellbeing and burnout among medical students in Wales. Int Rev Psychiatry. 2019;31(7–8):613–8. doi: 10.1080/09540261.2019.1678251 31638446

[34] Slavin SJ, Schindler DL, Chibnall JT. Medical student mental health 3.0: improving student wellness through curricular changes. Acad Med. 2014;89(4):573–7. doi: 10.1097/ACM.0000000000000166 24556765 PMC4885556

[35] Almaqbali M. Well-being among medical students in clinical years at a private college in Oman: cross sectional study. EC Psychol Psych. 2019;8:1129–35.

[36] Bloodgood RA, Short JG, Jackson JM, Martindale JR. A change to pass/fail grading in the first two years at one medical school results in improved psychological well-being. Acad Med. 2009;84(5):655–62. doi: 10.1097/ACM.0b013e31819f6d78 19704204

[37] Machado L, de Oliveira IR, Peregrino A, Cantilino A. Common mental disorders and subjective well-being: emotional training among medical students based on positive psychology. PLoS One. 2019;14(2):e0211926. doi: 10.1371/journal.pone.0211926 30731006 PMC6366695

[38] Angkurawaranon C, Jiraporncharoen W, Sachdev A, Wisetborisut A, Jangiam W, Uaphanthasath R. Predictors of quality of life of medical students and a comparison with quality of life of adult health care workers in Thailand. Springerplus. 2016;5(1):584. 27247881 10.1186/s40064-016-2267-5 PMC4864787

[39] Braun V, Clarke V. Using thematic analysis in psychology. Qual Res Psychol. 2006;3(2):77–101. doi: 10.1191/1478088706qp063oa

[40] Zigmond AS, Snaith RP. The hospital anxiety and depression scale. Acta Psychiatr Scand. 1983;67(6):361–70. doi: 10.1111/j.1600-0447.1983.tb09716.x 6880820

[41] Brennan C, Worrall-Davies A, McMillan D, Gilbody S, House A. The hospital anxiety and depression scale: a diagnostic meta-analysis of case-finding ability. J Psychosom Res. 2010;69(4):371–8. doi: 10.1016/j.jpsychores.2010.04.006 20846538

[42] Bjelland I, Dahl AA, Haug TT, Neckelmann D. The validity of the hospital anxiety and depression scale. An updated literature review. J Psychosom Res. 2002;52(2):69–77. doi: 10.1016/s0022-3999(01)00296-3 11832252

[43] Green MJ, Benzeval M. Ageing, social class and common mental disorders: longitudinal evidence from three cohorts in the West of Scotland. Psychol Med. 2011;41(3):565–74. doi: 10.1017/S0033291710000851 20444309 PMC3033734

[44] Newbury-Birch D, Lowry RJ, Kamali F. The changing patterns of drinking, illicit drug use, stress, anxiety and depression in dental students in a UK dental school: a longitudinal study. Br Dent J. 2002;192(11):646–9. doi: 10.1038/sj.bdj.4801448 12108944

[45] Marfell NR. Measuring depression and anxiety in medical students: Is HADS an appropriate tool? Cardiff University. 2019.

[46] Cohen S, Kamarck T, Mermelstein R. A global measure of perceived stress. J Health Soc Behav. 1983;24(4):385–96. 6668417

[47] Andreou E, Alexopoulos EC, Lionis C, Varvogli L, Gnardellis C, Chrousos GP, et al. Perceived stress scale: reliability and validity study in Greece. Int J Environ Res Public Health. 2011;8(8):3287–98. doi: 10.3390/ijerph8083287 21909307 PMC3166743

[48] Diener E, Emmons RA, Larsen RJ, Griffin S. The satisfaction with life scale. J Pers Assess. 1985;49(1):71–5. doi: 10.1207/s15327752jpa4901_13 16367493

[49] John O, Srivastava S. The big-five trait taxonomy: history, measurement, and theoretical perspectives. 1999.

[50] Carver CS. You want to measure coping but your protocol’s too long: consider the brief COPE. Int J Behav Med. 1997;4(1):92–100. doi: 10.1207/s15327558ijbm0401_6 16250744

[51] Pallant J. SPSS survival manual: a step by step guide to data analysis using IBM SPSS: McGraw-hill education (UK). 2020.

[52] Elliott AC, Woodward WA. Statistical analysis quick reference guidebook: with SPSS examples: Sage. 2007.

[53] Creswell JW. A concise introduction to mixed methods research/ John W. Creswell, University of Nebraska-Lincoln. Los Angeles: SAGE; 2015.

[54] Dawadi S, Shrestha S, Giri RA. Mixed-methods research: a discussion on its types, challenges, and criticisms. JPSE. 2021;2(2):25–36. doi: 10.46809/jpse.v2i2.20

[55] Melaku L, Bulcha G. Evaluation and comparison of medical students stressors and coping strategies among undergraduate preclinical and clinical year students enrolled in medical school of Arsi University, Southeast Ethiopia. Educ Res Int. 2021;2021.

[56] Ismail M, Lee KY, Sutrisno Tanjung A, Ahmad Jelani IA, Abdul Latiff R, Abdul Razak H, et al. The prevalence of psychological distress and its association with coping strategies among medical interns in Malaysia: a national‐level cross‐sectional study. Asia Pac Psychiatry. 2021;13(2):e12417. 10.1111/appy.12417 PMC8243927 32964660

[57] Wang Q, Sun W, Wu H. Associations between academic burnout, resilience and life satisfaction among medical students: a three-wave longitudinal study. BMC Med Educ. 2022;22(1):248. doi: 10.1186/s12909-022-03326-6 35382810 PMC8980514

[58] Quek TT-C, Tam WW-S, Tran BX, Zhang M, Zhang Z, Ho CS-H, et al. The global prevalence of anxiety among medical students: a meta-analysis. Int J Environ Res Public Health. 2019;16(15):2735. doi: 10.3390/ijerph16152735 31370266 PMC6696211

[59] Weber J, Skodda S, Muth T, Angerer P, Loerbroks A. Stressors and resources related to academic studies and improvements suggested by medical students: a qualitative study. BMC Med Educ. 2019;19(1):312. doi: 10.1186/s12909-019-1747-z 31429744 PMC6701044

[60] Abouammah N, Irfan F, Marwa I, Zakria N, AlFaris E. Stress among medical students and its consequences on health: a qualitative study. Biomed Res. 2020;31(1):1–8.

[61] Heinen I, Bullinger M, Kocalevent R-D. Perceived stress in first year medical students—associations with personal resources and emotional distress. BMC Med Educ. 2017;17(1):4. doi: 10.1186/s12909-016-0841-8 28056972 PMC5216588

[62] Mittal R, Su L, Jain R. COVID-19 mental health consequences on medical students worldwide. J Community Hosp Intern Med Perspect. 2021;11(3):296–8. doi: 10.1080/20009666.2021.1918475 34234896 PMC8118449

[63] Dost S, Hossain A, Shehab M, Abdelwahed A, Al-Nusair L. Perceptions of medical students towards online teaching during the COVID-19 pandemic: a national cross-sectional survey of 2721 UK medical students. BMJ Open. 2020;10(11):e042378. doi: 10.1136/bmjopen-2020-042378 33154063 PMC7646323

[64] Benoit V, Gabola P. Effects of positive psychology interventions on the well-being of young children: a systematic literature review. Int J Environ Res Public Health. 2021;18(22):12065. 34831827 10.3390/ijerph182212065 PMC8623229

[65] Seligman M. Evidence-based approaches in positive education: implementing a strategic framework for well-being in schools. Springer. 2015.

[66] Dyrbye LN, Harper W, Moutier C, Durning SJ, Power DV, Massie FS, et al. A multi-institutional study exploring the impact of positive mental health on medical students’ professionalism in an era of high burnout. Acad Med. 2012;87(8):1024–31. doi: 10.1097/ACM.0b013e31825cfa35 22722352

[67] MacArthur KR, Sikorski J. A qualitative analysis of the coping reservoir model of pre-clinical medical student well-being: human connection as making it “worth it”. BMC Med Educ. 2020;20(1):157. doi: 10.1186/s12909-020-02067-8 32429893 PMC7236216

[68] Thompson G, McBride RB, Hosford CC, Halaas G. Resilience among medical students: the role of coping style and social support. Teach Learn Med. 2016;28(2):174–82. doi: 10.1080/10401334.2016.1146611 27064719

[69] Moffat KJ, McConnachie A, Ross S, Morrison JM. First year medical student stress and coping in a problem-based learning medical curriculum. Med Educ. 2004;38(5):482–91. doi: 10.1046/j.1365-2929.2004.01814.x 15107082

[70] Bouteyre E, Maurel M, Bernaud JL. Daily hassles and depressive symptoms among first year psychology students in France: the role of coping and social support. Stress Health. 2007;23(2):93–9.

[71] Rafnsson FD, Jonsson FH, Windle M. Coping strategies, stressful life events, problem behaviors, and depressed affect. Anxiety Stress Coping. 2006;19(3):241–57. doi: 10.1080/10615800600679111

[72] Neufeld A, Malin G. How medical students cope with stress: a cross-sectional look at strategies and their sociodemographic antecedents. BMC Med Educ. 2021;21(1):299. doi: 10.1186/s12909-021-02734-4 34034732 PMC8152145

[73] McKinley N, McCain RS, Convie L, Clarke M, Dempster M, Campbell WJ, et al. Resilience, burnout and coping mechanisms in UK doctors: a cross-sectional study. BMJ Open. 2020;10(1):e031765. doi: 10.1136/bmjopen-2019-031765 31988223 PMC7045750

[74] Malpass A, Binnie K, Robson L. Medical students’ experience of mindfulness training in the UK: well-being, coping reserve, and professional development. Educ Res Int. 2019;2019. 10.1155/2019/4021729 PMC6546595 31168420

[75] Guthrie EA, Black D, Shaw CM, Hamilton J, Creed FH, Tomenson B. Embarking upon a medical career: psychological morbidity in first year medical students. Med Educ. 1995;29(5):337–41. doi: 10.1111/j.1365-2923.1995.tb00022.x 8699970

[76] Dunham L, Dekhtyar M, Gruener G, CichoskiKelly E, Deitz J, Elliott D, et al. Medical student perceptions of the learning environment in medical school change as students transition to clinical training in undergraduate medical school. Teach Learn Med. 2017;29(4):383–91. doi: 10.1080/10401334.2017.1297712 28318319

[77] van der Merwe LJ, Botha A, Joubert G. Resilience and coping strategies of undergraduate medical students at the University of the Free State. S Afr J Psychiatr. 2020;26:1471. doi: 10.4102/sajpsychiatry.v26i0.1471 32832128 PMC7433285

[78] Peleias M, Tempski P, Paro HB, Perotta B, Mayer FB, Enns SC, et al. Leisure time physical activity and quality of life in medical students: results from a multicentre study. BMJ Open Sport Exerc Med. 2017;3(1):e000213. doi: 10.1136/bmjsem-2016-000213 28761706 PMC5530174

[79] Dyrbye LN, Sciolla AF, Dekhtyar M, Rajasekaran S, Allgood JA, Rea M, et al. Medical school strategies to address student well-being: a national survey. Acad Med. 2019;94(6):861–8. doi: 10.1097/ACM.0000000000002611 30681453

[80] Sultan S, Labban OM, Hamawi AM, Alnajrani AK, Tawfik AM, Felemban MH, et al. Relationship of big five personality traits and future specialty preference among undergraduate medical students: a cross-sectional study. Egypt J Neurol Psychiatry Neurosurg. 2023;59(1). doi: 10.1186/s41983-023-00699-3

[81] Stienen MN, Scholtes F, Samuel R, Weil A, Weyerbrock A, Surbeck W. Different but similar: personality traits of surgeons and internists-results of a cross-sectional observational study. BMJ Open. 2018;8(7):e021310. doi: 10.1136/bmjopen-2017-021310 29982214 PMC6045716

[82] Mohamed Z, Jit Singh GK, Dediwadon NS, Mohamad Saleh NA, Jupri NN, Ganesan Y. Adult personality and its relationship with stress level, coping mechanism and academic performance among undergraduate nursing students. Malays J Med Sci. 2022;29(5):117–25. doi: 10.21315/mjms2022.29.5.12 36474539 PMC9680997

[83] Haider SI, Ahmed F, Pasha H, Pasha H, Farheen N, Zahid MT. Life satisfaction, resilience and coping mechanisms among medical students during COVID-19. PLoS One. 2022;17(10):e0275319. doi: 10.1371/journal.pone.0275319 36197934 PMC9534406

[84] Leszko M, Iwański R, Jarzębińska A. The relationship between personality traits and coping styles among first-time and recurrent prisoners in Poland. Front Psychol. 2020;10:2969. doi: 10.3389/fpsyg.2019.02969 32010025 PMC6972873

[85] Westerhof GJ, Keyes CLM. Mental illness and mental health: the two continua model across the lifespan. J Adult Dev. 2010;17(2):110–9. doi: 10.1007/s10804-009-9082-y 20502508 PMC2866965

