# Supplementary material for: Navigating the medical journey: Insights into medical students’ psychological wellbeing, coping, and personality
Source: PLoS One. 2025 Feb 6;20(2):e0318399. doi: 10.1371/journal.pone.0318399 (PMC11801719; doi:10.1371/journal.pone.0318399)

S1 Histogram and Q-Q Plot for Perceived Stress Scale, Hospital Anxiety Depression Scale, Satisfaction With Life Scale, Brief COPE and Big Five Inventory

Histogram and Q-Q Plot for Perceived Stress Scale-10 (PSS-10)


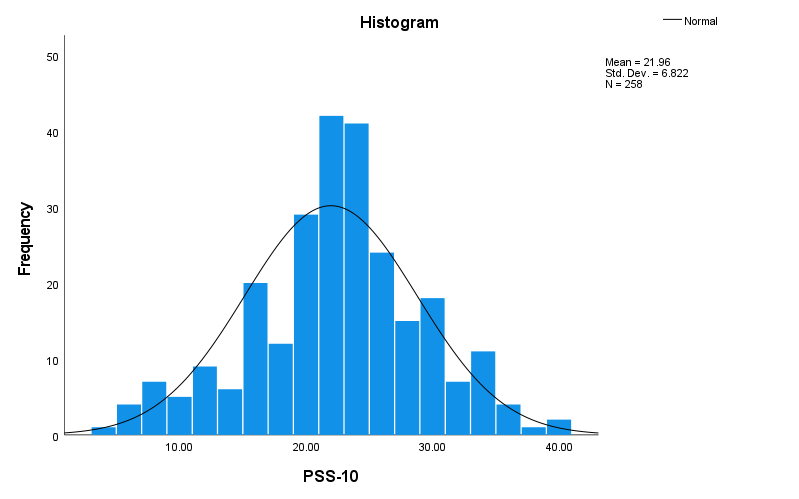


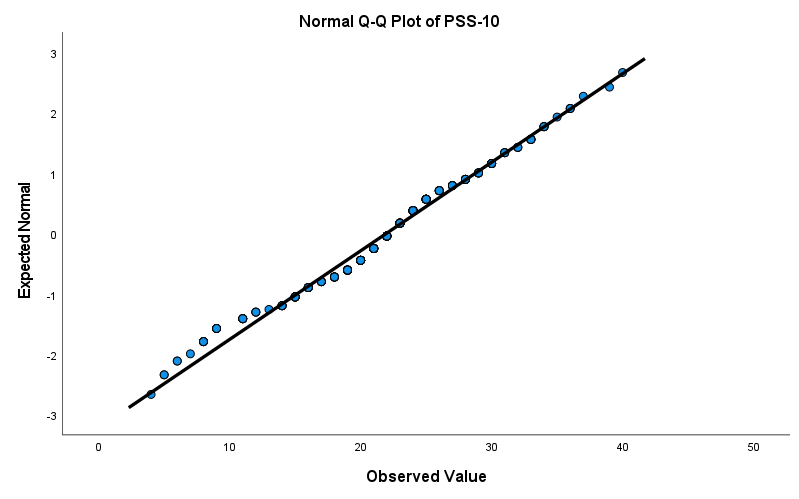


Histogram and Q-Q Plot for Hospital Anxiety Depression Scale (Depression)


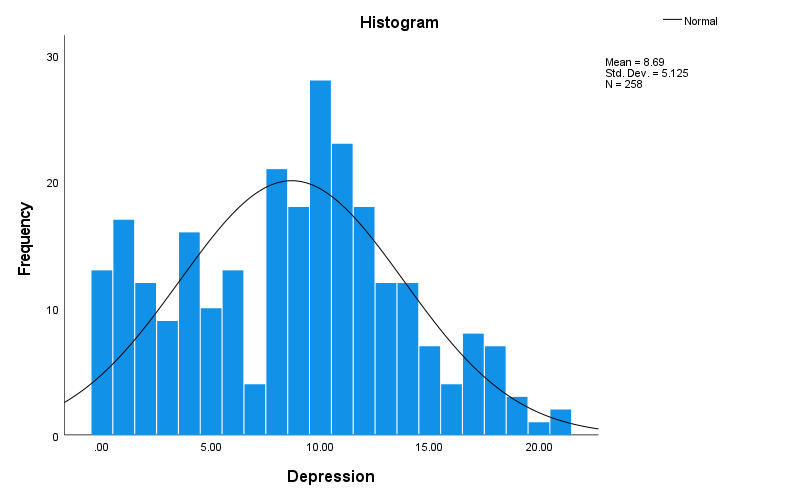

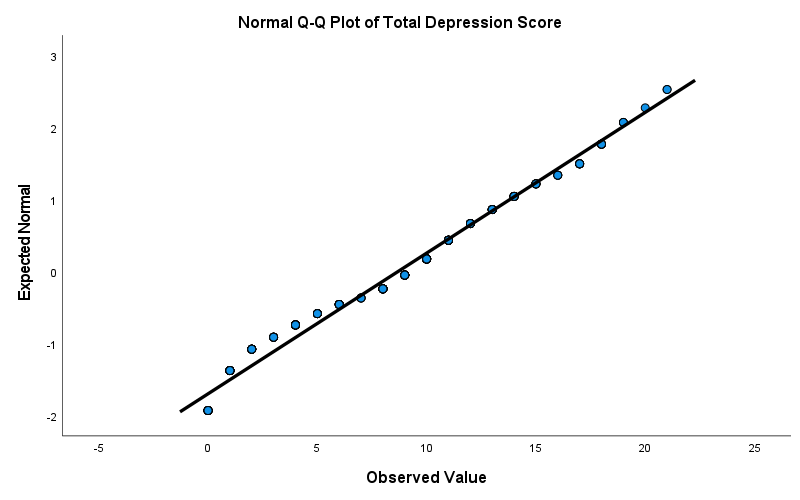


Histogram and Q-Q Plot for Hospital Anxiety Depression Scale (Anxiety)
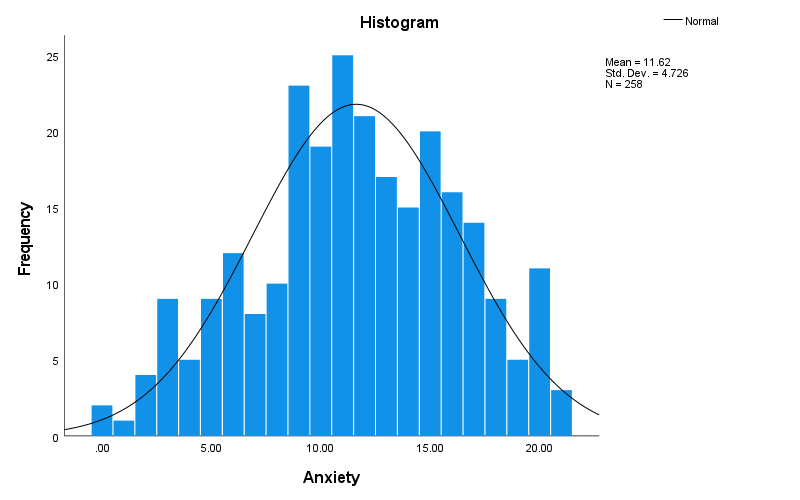

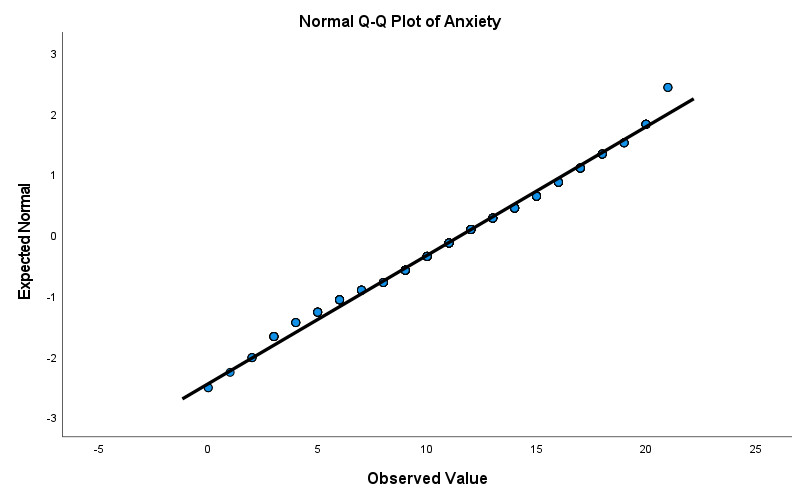


Histogram and Q-Q Plot for Satisfaction With Life Scale


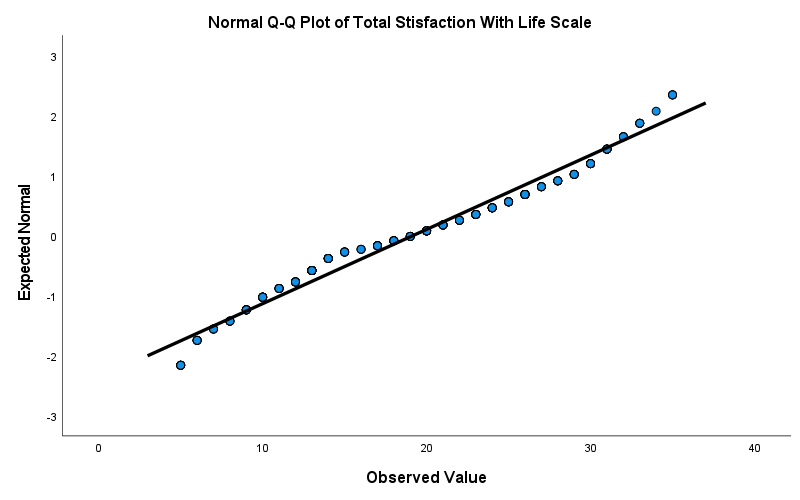

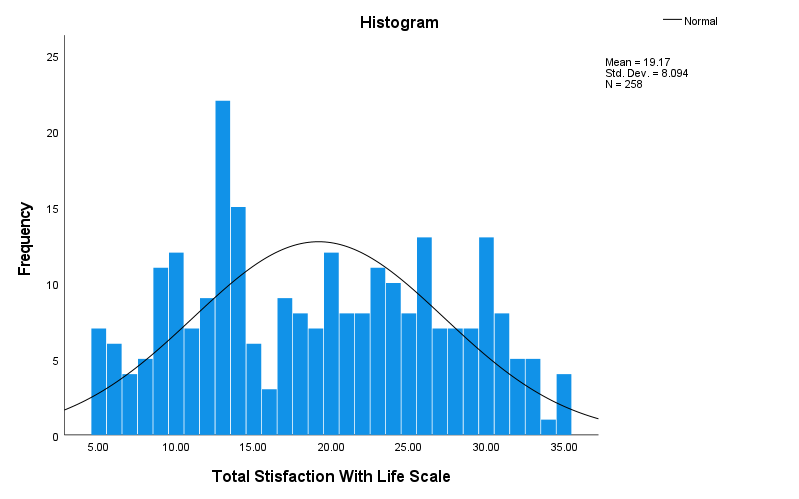


Histogram and Q-Q Plot for Brief COPE (Problem-Focused Coping)
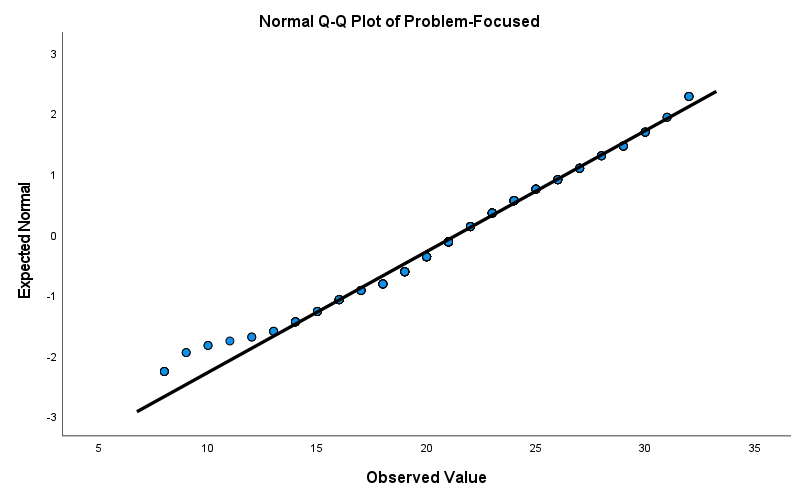

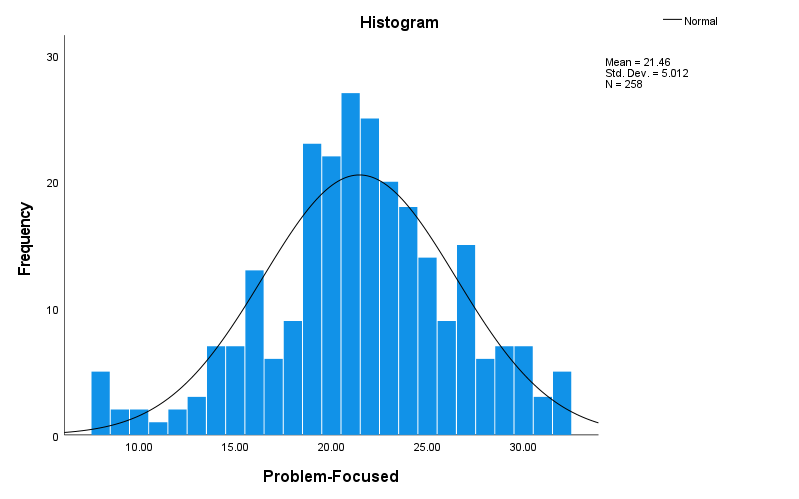


Histogram and Q-Q Plot for Brief COPE (Emotion-Focused Coping)


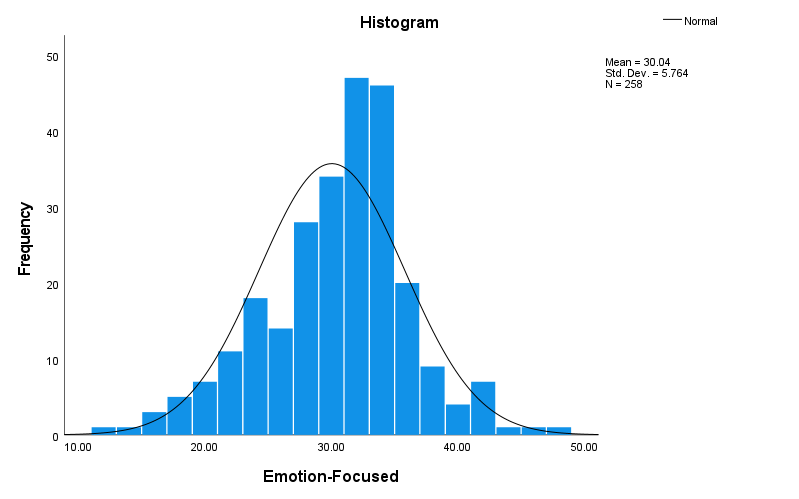

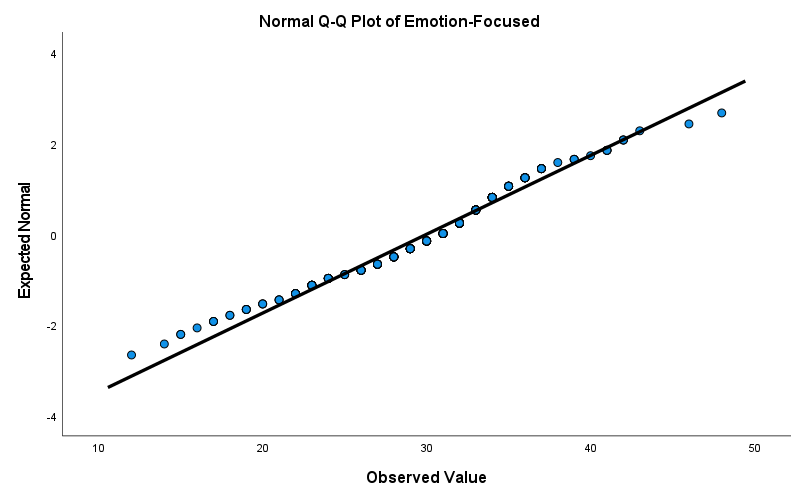


Histogram and Q-Q Plot for Brief COPE (Avoidance)
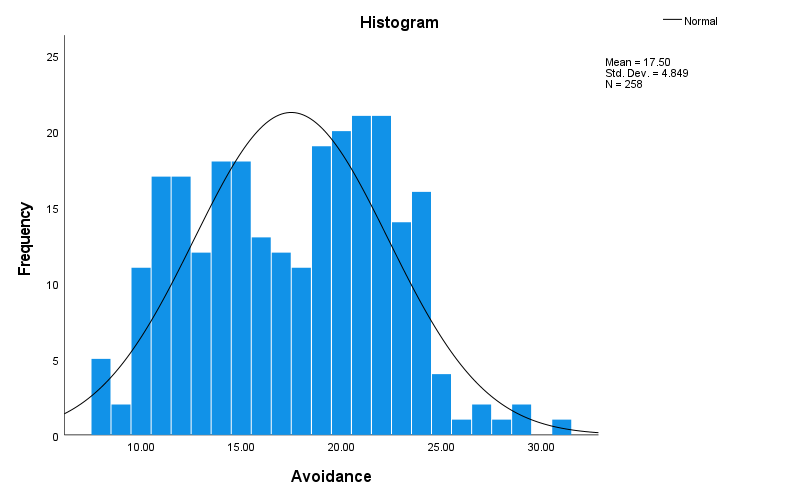

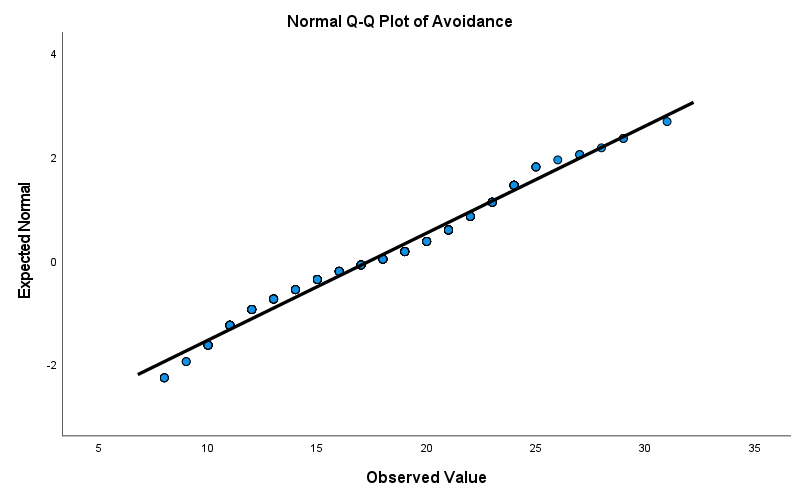


Q-Q Plot for Big Five Inventory (Extraversion & Agreeableness)
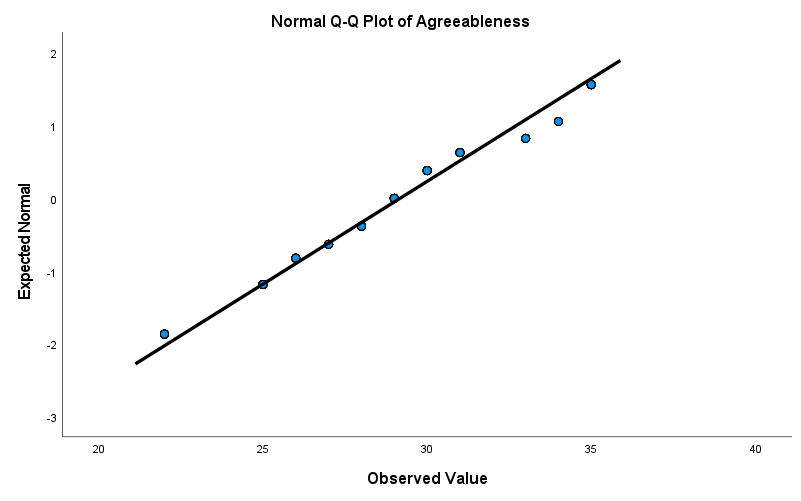

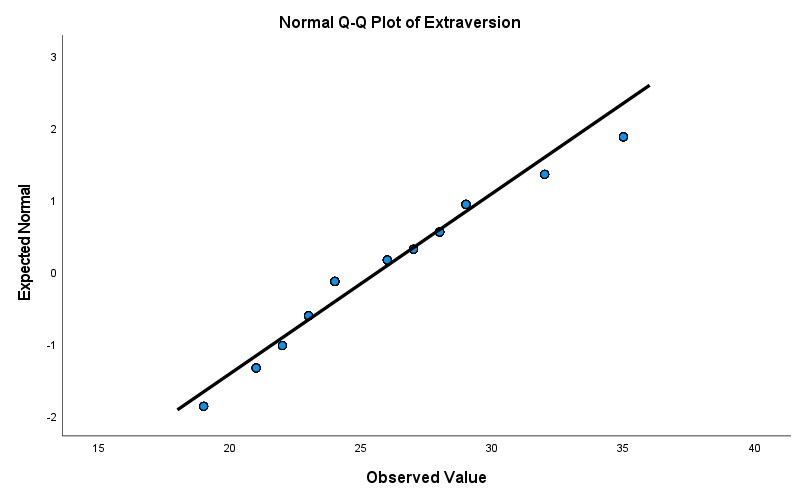


Q-Q Plot for Big Five Inventory (Conscientiousness, Neuroticism, Openness)


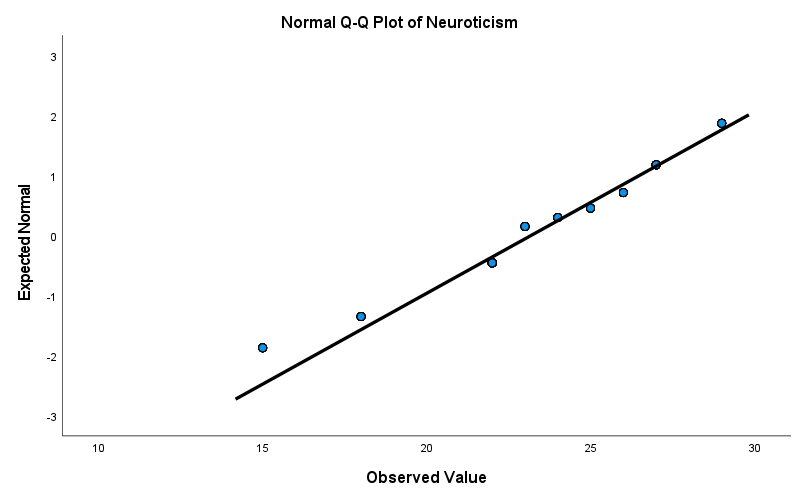

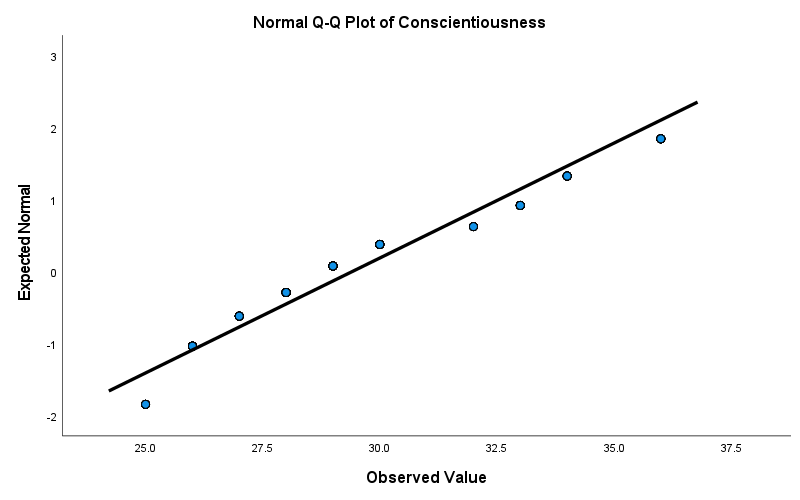


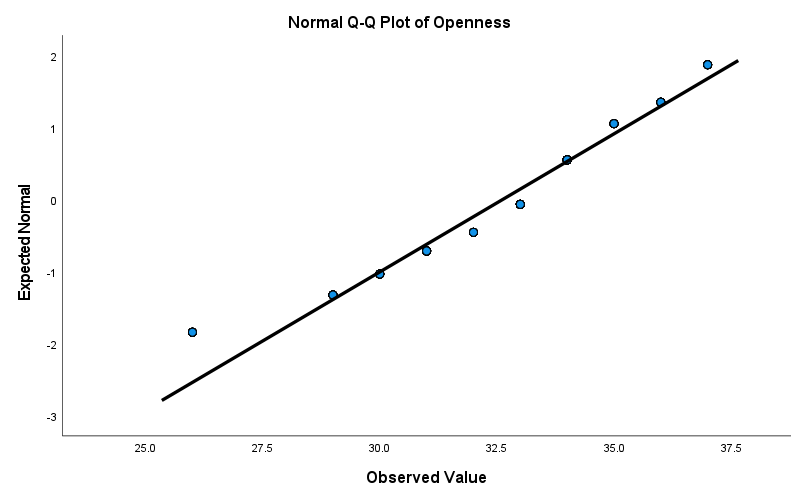

Supplement: S1 File — (DOCX) [file pone.0318399.s001.docx]
