# Supplementary material for: Navigating the medical journey: Insights into medical students’ psychological wellbeing, coping, and personality
Source: PLoS One. 2025 Feb 6;20(2):e0318399. doi: 10.1371/journal.pone.0318399 (PMC11801719; doi:10.1371/journal.pone.0318399)
Supplement: S2 File — (DOCX) [file pone.0318399.s002.docx]

**S2-Descriptions of themes 1-2 and their corresponding subthemes**

| Themes | Subthemes |
| --- | --- |
| Theme 1 - Perceived adverse academic, personal and organisational factors contribute to poor wellbeing | Subtheme 1a - Excessive workloads and exams concerns were sources of stress and low mood |
|  | Subtheme 1b - Adjusting to a new environment was challenging, stressful and emotionally heavy |
|  | Subtheme 1c - Students experience a lack of study-life balance due to increased workloads |
|  | Subtheme 1d - The medical school learning environment (culture) and students' perceptions about self and the course were understood to be sources of stress |
|  | Subtheme 1e - Having a mental health disorder and financial concerns added an additional burden on medical students' ability to manage a stressful course |
| Theme 2 - During COVID-19, medical students, were impacted negatively at an academic, personal and social level | Subtheme 2a - Lack of effective communication within the medical school during COVID-19 caused significant distress |
|  | Subtheme 2b - Students felt more stressed and less prepared as COVID-19 has decreased the amount of their clinical training. |
|  | Subtheme 2c - COVID-19 resulted in reduced cooping potential and reduced motivations and social isolation |
