# Supplementary material for: Navigating the medical journey: Insights into medical students’ psychological wellbeing, coping, and personality
Source: PLoS One. 2025 Feb 6;20(2):e0318399. doi: 10.1371/journal.pone.0318399 (PMC11801719; doi:10.1371/journal.pone.0318399)
Supplement: S3 File — (DOCX) [file pone.0318399.s003.docx]

***S3-Category*** *1 Quotes****: Factors adversely impacting medical students' psychological wellbeing***

| **Quotes** | **Participants** |
| --- | --- |
| **Quote 1** "The amount of self-directed learning we have to do is just insane" | P11 F, Year 3 |
| **Quote 2** ''Because we have exams in the next like six, seven weeks, that is obviously quite a stressful thing, and our exams count for a big part of our final year ranking now" | P13, F, Year 4 |
| **Quote 3** "I think that there's more workload than other courses, and I think that comparison is hard not to make sometimes when you see people on other courses, having a lot more free time than you do" | P8, F, Year 3 |
| **Quote 4** '' I feel stressed out starting placement and seeing people that are poorly, all the time. I think you get a really skewed view of things I always feel like everybody's poorly in the whole world. So I think sometimes that that doesn't help. I think that's quite emotionally heavy'' | P8, F, Year 3 |
| **Quote 5** "It's a lot of work to do alongside clinical placement, and demanding in terms of time, energy, I do find that things add up gradually, and you think, oh I'll do that tomorrow, I'll do that and then find that you've got a massive to-do list. It just seems quite unrealistic to get it all done" | P1, F, Year 3 |
| **Quote 6** " I have to sacrifice things like my social life; like If I have to call friends or family from back home, I don't do it as often as I should" P24, F, Year 4 | P24, F, Year 4 |
| **Quote 7** "There are things that are going better in my life …..but medical courses are a source of sadness and frustration, and it's also affecting the other good things that are going well" | P15, M, Year 4 |
| **Quote 8** if you hear the term, a gunner, like everyone's kind of medical students seem to be overachievers and that everyone's always doing all of the extracurricular things and there's a lot of pressure to be on all the committees and do all the sport and do loads of extra things" | P1, F, Year 3 |
| **Quote 9** ''I think the problem is the type of people that medicine attracts—all of the people that are predisposed to struggle to have that balance. I think medicine attracts people that are quite perfectionists and quite hard working. And I also think because there's so much work involved in getting even onto the course" | P8, F, Year 3 |
| **Quote 10** "Depression doesn't just affect your academic life. I was still struggling with the repercussions of being very disengaged personally, academically, and Socially for a period of time. So it definitely had an impact on everything" | P10, F, Year 4 |
| **Quote 11**  "During the pandemic I think academically, there was a lot of stress with the medical school organisation " | P25, M, Year 4 |
| **Quote 12**  ''The balance was skewed, very much towards the academic life, instead of personal life before COVID, and since COVID, It got skewed even more''  ''COVID has made my life harder, it took away six months of placement, which I think are vital, and I think, by not having them, I'm behind where I should be'' | P15, M, Year 4 & 12, P7, F, Year 4 |
| **Quote 13** "With so much of our studies moving online. I felt a little bit demotivated. Yeah, I ended up not being as productive as a result'' | P4, F, Year 5 |
| **Quote 14** "[ During COVID] I've not been able to exercise as much, and as a result I've sort of become less healthy" | P2, M, Year 5 |
| **Quote 15** ''Basically during COVID time, I suppose, at some points when I'm at the shops, there's lots of people that are not staying away from me. I get stressed about that a lot. So I suppose the way that I deal with that is, try to kind of slow down and I can't, it's something I can't control so I have to just kind of step back and try not to let it over like get too into my head'' | P9, F, Year 2 |
| **Quote 16** "I'm not sure because it's not clear cut. The first two years were accelerated, condensing 18 months of content into two and a half years. Those initial 18 months were stressful, contemplating the need to learn extensively; compounded by difficulties with their exam style. However, all of that was within your control to improve or worsen, depending on how much you work. In contrast, with COVID, so much was out of your control. I kinda was like, Well, I can't do anything about it." "[During COVID-19] it feels a lot more lonely, a lot more isolated because you've not got all of these different connections" | P20, F, Year 3  P16, F, Year 3 |
