# Supplementary material for: Navigating the medical journey: Insights into medical students’ psychological wellbeing, coping, and personality
Source: PLoS One. 2025 Feb 6;20(2):e0318399. doi: 10.1371/journal.pone.0318399 (PMC11801719; doi:10.1371/journal.pone.0318399)
Supplement: S4 File — (DOCX) [file pone.0318399.s004.docx]

**S4-Descriptions of themes 3-4 and their corresponding subthemes**

| Themes | Subthemes |
| --- | --- |
| Theme 3 – Certain aspects relevant to the medical school course and study-life balance were associated with a positive mood | Subtheme 3a - Managing work whilst also allowing for leisure time helped maintain a long-term positive perspective on (academic) life |
|  | Subtheme 3b - A sense of achievement was reported as a good feeling that boosts mood |
|  | Subtheme 3c - Direct contact with patients and professional support made medical students feel helpful and fulfilling |
|  | Subtheme 3d - Meaningful relationships with friends and family and peer socialising brought about a sense of happiness |
| Theme 4 - COVID-19 had some positive impacts on an academic and personal level | Subtheme 4a - Online learning was found to be easier, with accessibility to the recorded lectures at any time |
|  | Subtheme 4b - Developing new hobbies that could be sustained was deemed to be positive |
