# Supplementary material for: Navigating the medical journey: Insights into medical students’ psychological wellbeing, coping, and personality
Source: PLoS One. 2025 Feb 6;20(2):e0318399. doi: 10.1371/journal.pone.0318399 (PMC11801719; doi:10.1371/journal.pone.0318399)
Supplement: S5 File — (DOCX) [file pone.0318399.s005.docx]

***S5-Category 2*** *Quotes****: Factors positively impacting medical students' psychological wellbeing***

| **Quotes** | **Participants** |
| --- | --- |
| **Quote 17** "I'm better at setting boundaries and kind of say, right, I'll work this kind of period of time and then I'll have this period of time to relax or chat with friends and family, which could help me for the long run" | P8, F, year 3 |
| **Quote 18** "I think there are also days on the ward where you get a really nice Teaching Fellow kind of reassures you and says, you know, you don't need to know that you don't need that much detail .. and at that point, it's a bit more of that perspective type of thing where you think okay like well I don't need to do that now. So .. I think the workload is quite a lot, so just being told that you don't have to worry about something, it's really nice" | P16, F, Year 3 |
| **Quote 19**  The times I felt happy is when I'm just hanging out with friends, it doesn't really matter where the air is like, you know, it's just in my living outside, it's just that, that's when I feel the most happy" room in my uni house, or, you know, | P5, F, Year 3 |
| **Quote 20 "**I think I'm finding my studies easier this time because things are recorded, and stuff is easy to keep up, and if I have a bad day, I can catch up later'' P9, F, Year 2 | P9, F, Year 2. |
