# Supplementary material for: Navigating the medical journey: Insights into medical students’ psychological wellbeing, coping, and personality
Source: PLoS One. 2025 Feb 6;20(2):e0318399. doi: 10.1371/journal.pone.0318399 (PMC11801719; doi:10.1371/journal.pone.0318399)
Supplement: S6 File — (DOCX) [file pone.0318399.s006.docx]

**S6-Descriptions of theme 5 and corresponding subthemes**

| Themes | Subthemes |
| --- | --- |
| Theme 5 - Adopting active strategies to manage stress and mood | Subtheme 5a- Keeping an activity planner and a daily routine can help in managing stress |
|  | Subtheme 5b - Extracurricular activities provided a way of relaxation and helped maintain a positive mood |
|  | Subtheme 5c - Taking the time to reach out to others or to reflect on the stressful situation |
