# Supplementary material for: Navigating the medical journey: Insights into medical students’ psychological wellbeing, coping, and personality
Source: PLoS One. 2025 Feb 6;20(2):e0318399. doi: 10.1371/journal.pone.0318399 (PMC11801719; doi:10.1371/journal.pone.0318399)
Supplement: S7 File — (DOCX) [file pone.0318399.s007.docx]

***S7-Category 3*** Quotes***: Coping strategies used to manage students' psychological wellbeing***

| **Quotes** | **Participants** |
| --- | --- |
| **Quote 21** "I like to journal about it. So just write, you know what happened, how I found what I learned like stuff like that because I think that just gets out my head a bit, and enables me to stretch my thoughts in a way that sort of helpful and relaxing..' I really think journaling helps and also giving it a bit of time, because like, sort of sleeping on it… and I think waking up has removed the sort of negative emotional rind of the situation if that makes sense'' | P5, F, Year 3 |
| **Quote 22** "I also really enjoy cooking, so try to make sure that I have nice meals in the evening so that I know I spend like a nice half an hour 40 minutes just cooking something because it takes my mind off everything that's going on completely distraction'' | P13, F, Year 4 |
| **Quote 23** "A bit of contextualising in compartmentalising; I've read a lot of good books, which talk about how well it works, but I think it's worked for me, sort of just taking a moment in your day or your week to just sit and talk out loud itself about what you're feeling at that point in time, and I've certainly found I've had to do that a lot more this year, and just rationalising things in your own head and setting goals, to move forward and accepting that you can't always take everything you want to off the list, there's always going to be something else" | "P2, M, Year 5 |
| **Quote 24** "I started placement that has meant like, I've had to get up with life that I am some days, I won't be happy, I would say; But otherwise, because we feel a bit uncomfortable sometimes on placement or a bit useless. It's just dealing with those sort of feelings and having to accept them." | P5, F, Year 3 |
